# Increased vascular smooth muscle cell senescence in aneurysmal *Fibulin-4* mutant mice

**DOI:** 10.1038/s41514-024-00154-4

**Published:** 2024-06-20

**Authors:** Sanne J. M. Stefens, Nicole van Vliet, Arne IJpma, Joyce Burger, Yunlei Li, Paula M. van Heijningen, Jan H. N. Lindeman, Danielle Majoor-Krakauer, Hence J. M. Verhagen, Roland Kanaar, Jeroen Essers, Ingrid van der Pluijm

**Affiliations:** 1 Department of Molecular Genetics, Erasmus University Medical Center, Rotterdam, The Netherlands; 2 Department of Pathology, Erasmus University Medical Center, Rotterdam, The Netherlands; 3 Department of Clinical Genetics, Erasmus University Medical Center, Rotterdam, The Netherlands; 4 Department of Vascular Surgery, Leiden University Medical Center, Leiden, The Netherlands; 5 Department of Vascular Surgery, Cardiovascular Institute, Erasmus University Medical Center, Rotterdam, The Netherlands; 6 Department of Radiotherapy, Erasmus University Medical Center, Rotterdam, The Netherlands; 7 Oncode Institute, Erasmus MC Cancer Institute, Erasmus University Medical Center, Rotterdam, Netherlands

**Keywords:** Cardiovascular diseases, Ageing, Senescence

## Abstract

Aortic aneurysms are dilatations of the aorta that can rupture when left untreated. We used the aneurysmal *Fibulin-4*^*R/R*^ mouse model to further unravel the underlying mechanisms of aneurysm formation. RNA sequencing of 3-month-old *Fibulin-4*^*R/R*^ aortas revealed significant upregulation of senescence-associated secretory phenotype (SASP) factors and key senescence factors, indicating the involvement of senescence. Analysis of aorta histology and of vascular smooth muscle cells (VSMCs) in vitro confirmed the senescent phenotype of *Fibulin-4*^*R/R*^ VSMCs by revealing increased SA-β-gal, p21, and p16 staining, increased IL-6 secretion, increased presence of DNA damage foci and increased nuclei size. Additionally, we found that p21 luminescence was increased in the dilated aorta of *Fibulin-4*^*R/R*^|p21-luciferase mice. Our studies identify a cellular aging cascade in Fibulin-4 aneurysmal disease, by revealing that *Fibulin-4*^*R/R*^ aortic VSMCs have a pronounced SASP and a senescent phenotype that may underlie aortic wall degeneration. Additionally, we demonstrated the therapeutic effect of JAK/STAT and TGF-β pathway inhibition, as well as senolytic treatment on *Fibulin-4*^*R/R*^ VSMCs in vitro. These findings can contribute to improved therapeutic options for aneurysmal disease aimed at reducing senescent cells.

## Introduction

Aortic aneurysms are pathological dilatations of the aorta to greater than 50% of its original size and can lead to dissections, which have a high mortality rate. Aortic aneurysms and dissections are responsible for 1–2% of all deaths in developed countries^1^. Next to advanced age, risk factors for developing aortic aneurysms include male gender and family history of aortic aneurysms^2^. Thoracic aortic aneurysms (TAA) are usually observed in inherited syndromes, such as Marfan syndrome, Loeys–Dietz syndrome, and cutis laxa syndrome. Heterozygous mutations in *FBN1*, the gene encoding for Fibrillin-1 protein involved in extracellular matrix (ECM) integrity, cause Marfan syndrome (MFS1; MIM 154700), a fibrous connective tissue disorder characterized by skeletal, ocular, and cardiovascular abnormalities including aneurysm development. Patients with mutations in the *SMAD2/3* and *TGFBR1/2* genes, encoding proteins involved in transforming growth factor beta (TGF-β) signaling, develop Loeys–Dietz syndrome (TGFBR1; LDS1; MIM 609192, TGFBR2; LDS2; MIM 61068, SMAD2; LDS6; MIM 619656, SMAD3; LDS3 (AOS); MIM 613795). This syndrome is characterized by aneurysms, dissections, and cardiac abnormalities, as well as early-onset osteoarthritis^3,4^. Homozygous or compound heterozygous mutations in *EFEMP2*, which encodes for the ECM protein Fibulin-4, have been found in patients with cutis laxa syndrome (ARCL1B; MIM 614437), which is characterized by loose skin, lung emphysema, bone fragility, vascular tortuosity and aneurysms^5,6^. Fibulin-4 is located in microfibril bundles that tether elastic fibers to vascular smooth muscle cells (VSMCs) and therefore plays an essential role in elastic fiber formation^7^. Fibulin-4 is particularly important in vascular maturation and the maintenance of structural integrity and elasticity of the aortic wall^8,9^.

Fibulin-4 deficiency has been mimicked in previously developed *Fibulin-4*^*R/R*^ mutant mice with a systemic 4-fold decreased expression of Fibulin-4^8^. *Fibulin-4*^*R/R*^ mice (where R stands for reduced expression) develop TAAs and additionally present with increased arterial stiffness and impaired vascular contractility^8^. *Fibulin-4*^*R/R*^ mice die at an early age of 3 months. Deregulation of the TGF-β signaling pathway, involved in the regulation of proliferation, differentiation, and development, underlies aortic aneurysm development in patients and mice with *Fibulin-4* deficiency. In the aorta of *Fibulin-4*^*R/R*^ mice, an increase in free TGF-β causes activation of downstream transcription of the TGF-β pathway, resulting in increased matrix metalloproteinase (MMP) activity and ECM remodeling^10–12^. Furthermore, mitochondrial dysfunction plays an important role in aneurysm formation both in *Fibulin-4*^*R/R*^ mice and aneurysm patients^9,13^.

Aging is a major risk factor for developing aortic aneurysms^2,14^. With age, many structural and functional changes occur in the aorta such as increased stiffness, increased aortic diameter, inflammation, aberrant ECM, and endothelial dysfunction^15^. More specifically, VSMCs, which are the main cell type of the aortic media, can undergo phenotypic changes under the influence of pathological stimuli such as those that occur with aging^16^. Many of these aforementioned processes are also involved in aortic aneurysm formation. Therefore, we aimed to investigate these processes in our aneurysmal *Fibulin-4*^*R/R*^ mouse model.

To further investigate the molecular mechanisms underlying aneurysm formation in *Fibulin-4*^*R/R*^ mice, RNA sequencing of the aorta was performed. RNA expression analysis confirmed previously identified deregulated molecular pathways such as the TGF-β pathway and pathways involved in ECM remodeling and mechanosensing. Additionally, data analysis pointed to the involvement of genes and pathways involved in senescence and VSMC phenotypic switching. Furthermore, we identified markers for the senescent phenotype in aneurysmal *Fibulin-4*^*R/R*^ aortic tissue and VSMCs and demonstrated the therapeutic effect of JAK/STAT and TGF-β pathway inhibition and senolytic treatment.

## Results

### RNAseq analysis confirms previously identified pathways involved in aneurysm formation in *Fibulin-4*^*R/R*^ mice

**Fig. 1:**
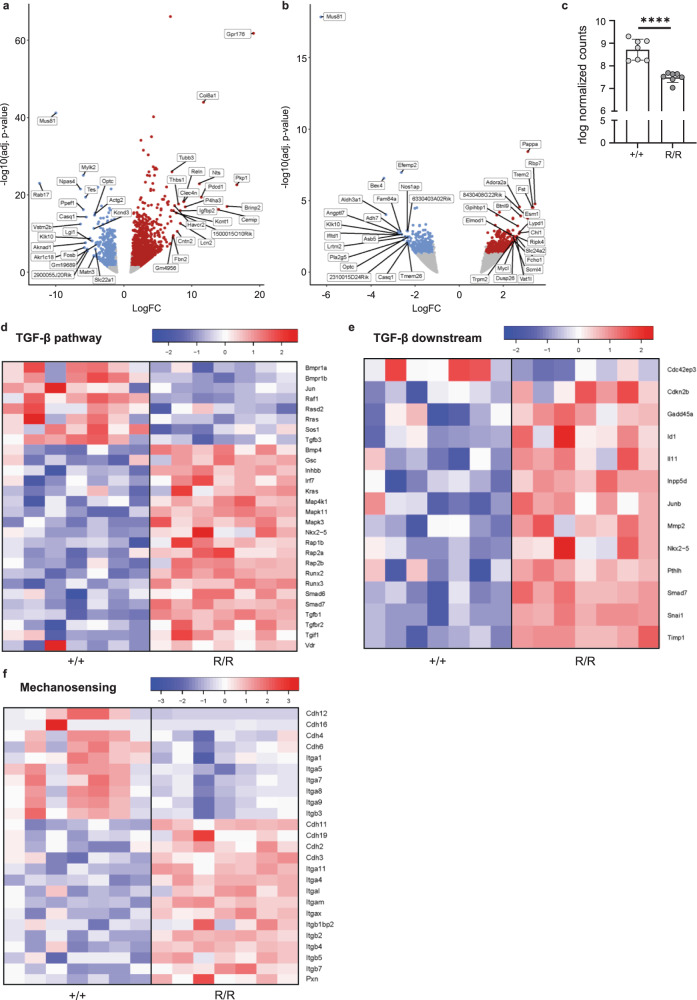
*Analysis of RNA sequencing data from the Fibulin-4 mutant mouse aorta.* **a** Volcano plot of genes differentially expressed between the *Fibulin-4*^*R/R*^ aortic arch and the *Fibulin-4*^*+/+*^ aortic arch. **b** Volcano plot of genes differentially expressed between the *Fibulin-4*^*R/R*^ abdominal aorta and the *Fibulin-4*^*+/+*^ abdominal aorta. The top 20 differentially expressed genes are highlighted (ranked based on fold change). Significantly upregulated genes are colored red and significantly downregulated genes are colored blue (*p* value ≤ 0.05; |fold change| ≥ 1.2). **c** Expression of the *Efemp2* gene (rlog normalized counts), encoding Fibulin-4 protein, in the *Fibulin-4*^*R/R*^ aortic arch compared to the *Fibulin-4*^*+/+*^ aortic arch (****p < 0.0001, FC = −2.421). The mean (±SD) is plotted. **d** Heatmap of significantly differentially expressed genes in the TGF-β pathway (*p* ≤ 0.05). **e** Heatmap of significantly differentially expressed genes downstream of the TGF-β pathway (*p* ≤ 0.05). **f** Heatmap of significantly differentially expressed genes involved in mechanosensing (*p* ≤ 0.05).

To further investigate the molecular mechanisms underlying aortic aneurysm formation in *Fibulin-4*^*R/R*^ mice, RNAseq was performed on *Fibulin-4*^*R/R*^ mouse aorta using the Illumina NextSeq500 platform. Differential expression analysis resulted in 3036 significant differentially expressed genes (*p* value ≤ 0.05; |fold change| ≥ 1.2) in the aortic arch (Fig. 1a and Supplementary Table 1) and 851 in the abdominal aorta (Fig. 1b and Supplementary Table 1) of *Fibulin-4*^*R/R*^ mice compared to *Fibulin-4*^*+/+*^ mice. *Fibulin-4*^*R/R*^ mice develop an aneurysm in the aortic arch, possibly resulting in more differentially expressed genes in the aortic arch compared to the abdominal aorta, which is less affected. Therefore, we focused on the aortic arch in this study. The differential expression data confirmed that expression of the gene for Fibulin-4, *Efemp2*, was significantly downregulated in the *Fibulin-4*^*R/R*^ aorta (Fig. 1c).

Since the *Fibulin-4*^*R/R*^ mouse model was developed to study aneurysm formation, several pathways have been uncovered to play a role in disease development in these mice. The TGF-β signaling pathway is the main pathway known to be involved in aneurysm formation in patients and mice with decreased Fibulin-4 expression^6,10,11^. Therefore, we investigated whether RNA expression analysis of *Fibulin-4*^*R/R*^ mouse aorta would also reveal an increase in TGF-β pathway activity using Ingenuity Pathway Analysis (IPA). Our data showed significant deregulation of the TGF-β signaling pathway (−log (*p* value) > 1.3) in the *Fibulin-4*^*R/R*^ aortic arch without clear directionality (*z* score ≤ 2)(Fig. 1d and Supplementary Fig. 1a). Nonetheless, upregulation of downstream genes *Gsc*, *Smad6*, *Smad7*, *Irf7*, and *Nkx2.5* was observed. Additionally, analysis of 26 target genes of the TGF-β pathway (a list previously generated to determine pathway activity based on downstream gene expression^17^) revealed that 46.2% of analyzed target genes (12/26 genes) were upregulated in the *Fibulin-4*^*R/R*^ aortic arch, whereas only one gene was downregulated (Fig. 1e and Supplementary Fig. 1b). Thus, our RNA expression data from 3-month-old animals was in line with previous findings in 10-day-old animals and suggested increased TGF-β signaling in the aortic arch of *Fibulin-4*^*R/R*^ mice^8^.

Our RNAseq analysis revealed that in the *Fibulin-4*^*R/R*^ aortic arch, integrin signaling, integrin-like kinase (ILK) signaling, calcium signaling, RhoA mediated signaling, regulation of actin-based motility by Rho, actin cytoskeleton signaling and focal adhesion kinase (FAK) signaling were significantly changed (Supplementary Table 2). More specifically, the expression of important mechanosensing protein-encoding genes such as cadherins, integrins, and paxillin was significantly changed in the *Fibulin-4*^*R/R*^ aortic arch (Fig. 1f). These results confirmed previous findings in *Fibulin-4*^*SMKO*^ mice (SMKO = smooth muscle cell knockout), revealing that alterations in the structure and mechanical properties of the aortic wall are associated with dysregulated mechanosensing in *Fibulin-4*^*SMKO*^ VSMCs^18^. These results suggest that mechanosensing is dysregulated in the aorta of mice with decreased Fibulin-4 expression.

### Cytoskeleton reorganization and ECM remodeling in aortas of *Fibulin-4*^*R/R*^ mice

**Fig. 2:**
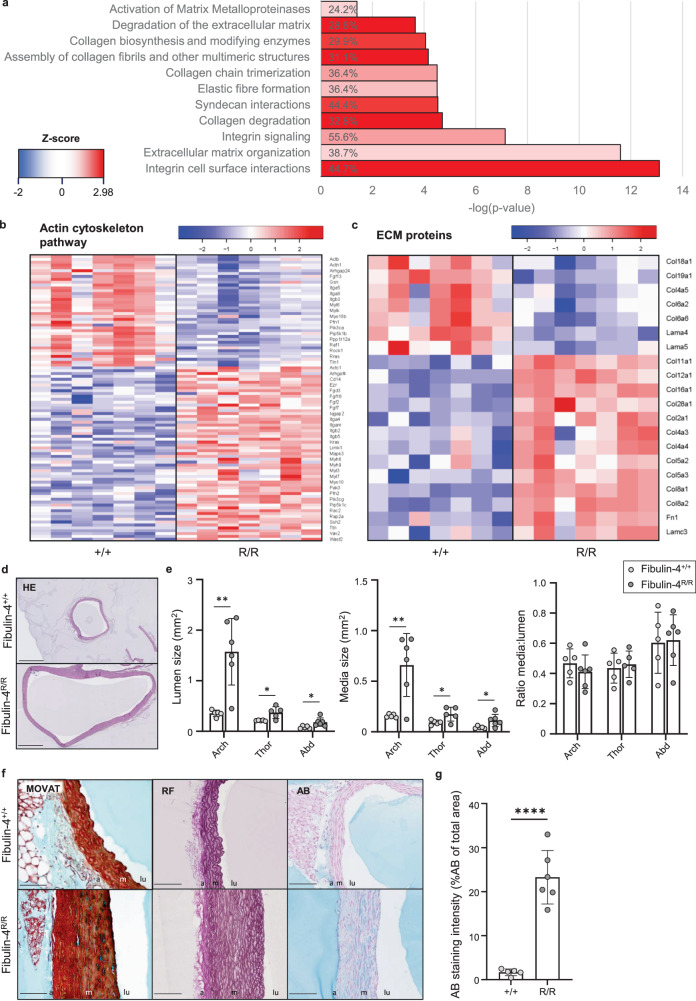
Cytoskeleton reorganization and ECM remodeling in the Fibulin-4 mutant mouse aorta. **a** Significantly changed canonical pathways involved in ECM remodeling from IPA analysis (−log (*p* value) > 1.3). Percentages written in the bars represent the ratio ((number of genes in the pathway that meet cutoff criteria/total number of genes in the pathway)* 100%). **b** Heatmap of significantly differentially expressed genes in the actin cytoskeleton pathway (*p* ≤ 0.05). Note: not all gene names were plotted. **c** Heatmap of significantly differentially expressed ECM proteins in the *Fibulin-4*^*R/R*^ aortic arch compared to *Fibulin-4*^*+/+*^controls (*p* ≤ 0.05). **d** HE staining of *Fibulin-4*^*R/R*^ and *Fibulin-4*^*+/+*^ mouse aortic arch. Bar = 500 µm. **e** Quantification of the lumen size, media size, and media:lumen ratio. In all graphs, the mean (±SD) is plotted (*n* = 5–6 per group, *p < 0.05, **p < 0.01, unpaired *t* test). Arch aortic arch, Thor thoracic aorta, Abd abdominal aorta. **f** Movat’s pentachrome staining (left) of *Fibulin-4*^*R/R*^ mouse aorta identifying collagen (yellow) and fibrin (bright red) deposition, elastin fiber (black) disorganization and proteoglycan (blue) deposition at sites of smooth muscle cell (red) loss compared to *Fibulin-4*^*+/+*^ controls (*n* = 3 per group). Resorcin Fuchsin staining (middle) of *Fibulin-4*^*R/R*^ and *Fibulin-4*^*+/+*^ mouse aortas (*n* = 5–6 per group). Alcian blue staining (right) of *Fibulin-4*^*R/R*^ and *Fibulin-4*^*+/+*^ mouse aortas (*n* = 5–6 per group). Bar = 100 mm, m media, a adventitia, lu lumen. **g** Quantification of Alcian blue staining in the *Fibulin-4*^*R/R*^ and *Fibulin-4*^*+/+*^ aortic wall. The mean percentage (±SD) of Alcian blue staining of the total aortic media surface area is plotted (*n* = 5–6 per group, ****p < 0.0001, unpaired *t* test).

Previous research showed that *Fibulin-4*^*R/R*^ VSMCs present with excessive ECM production and aberrations in actin cytoskeleton structure and dynamics^10^. The formation and proper functioning of the cytoskeleton is essential for VSMC contractility. Additionally, VSMCs are highly sensitive to changes of the surrounding ECM, which can therefore also influence their state. Our RNA expression analysis also revealed several significantly changed genes and pathways that are associated with cytoskeleton reorganization and ECM production (Fig. 2a). The actin cytoskeleton pathway was the fourth most significantly changed pathway in the *Fibulin-4*^*R/R*^ aortic arch and was predicted to be upregulated with a *z* score of 2.32 (Fig. 2b and Supplementary Fig. 1c). The top 20 of upregulated genes contained several genes encoding structural proteins, such as collagen type VIII alpha 1 chain (*Col8a1*), reelin (*Reln*), thrombospondin 1 (*Thbs1*), fibrillin 2 (*Fbn2*), and tubulin beta 3 class III (*Tubb3*) (Fig. 1a). Additionally, expression of 15 other collagen chains, 3 laminin subunits and fibronectin 1 (*Fn1*) was significantly changed, all of which are ECM proteins (Fig. 2c). The genes encoding for myosin light chain kinase 2 (*Mylk2*) and actin gamma 2 (*Actg2*), proteins involved in skeletal muscle contraction, were in the top 20 downregulated genes in the *Fibulin-4*^*R/R*^ aortic arch (Fig. 1a). These results suggested that dysregulation of mechanisms involved in the maintenance of proper aortic wall structure and function is reflected at the RNA expression level.

To validate findings from the RNAseq data analysis, histochemical stainings for several (extra) cellular structures were performed on the mouse aorta. In *Fibulin-4*^*R/R*^ mice, media and lumen size were significantly increased in the aortic arch (Fig. 2d, e). No significant change was observed in the ratio between media and lumen, indicating that the increase in media size is equivalent to the increase in lumen size in the *Fibulin-4*^*R/R*^ mouse aorta (Fig. 2e). Furthermore, Movat’s staining revealed fibrotic changes portrayed by increased collagen (yellow) and fibrin (bright red) deposition, as well as disorganization of elastin fibers (black) in the medial layer of the *Fibulin-4*^*R/R*^ aorta (Fig. 2f). Additionally, proteoglycan (blue) deposition was visible particularly at sites with loss of smooth muscle cells (red). RNAseq data analysis revealed significantly changed genes and pathways involved in ECM remodeling in the *Fibulin-4*^*R/R*^ aortic arch. Elastin is one of the main components of the ECM involved in pathological remodeling of the aortic wall upon aneurysm formation. Resorcin-fuchsin (RF) staining confirmed altered elastin structures in the *Fibulin-4*^*R/R*^ aorta (Fig. 2f). Accumulation but also disorganization of elastic laminae was visible in the media of the *Fibulin-4*^*R/R*^ aorta. Additionally, flattening of the elastic laminae was observed and further reflected stiffening of the aortic wall^19,20^. Alcian blue staining showed a significantly increased accumulation of proteoglycans, another major component of the ECM, in the aortic wall of *Fibulin-4*^*R/R*^ mice (Fig. 2f, g). These findings together confirmed our RNAseq data, showing significant ECM remodeling in the aortic wall of *Fibulin-4*^*R/R*^ mice.

### Significantly altered expression of markers for the contractile and synthetic VSMC phenotype in *Fibulin-4*^*R/R*^ aortas

**Fig. 3:**
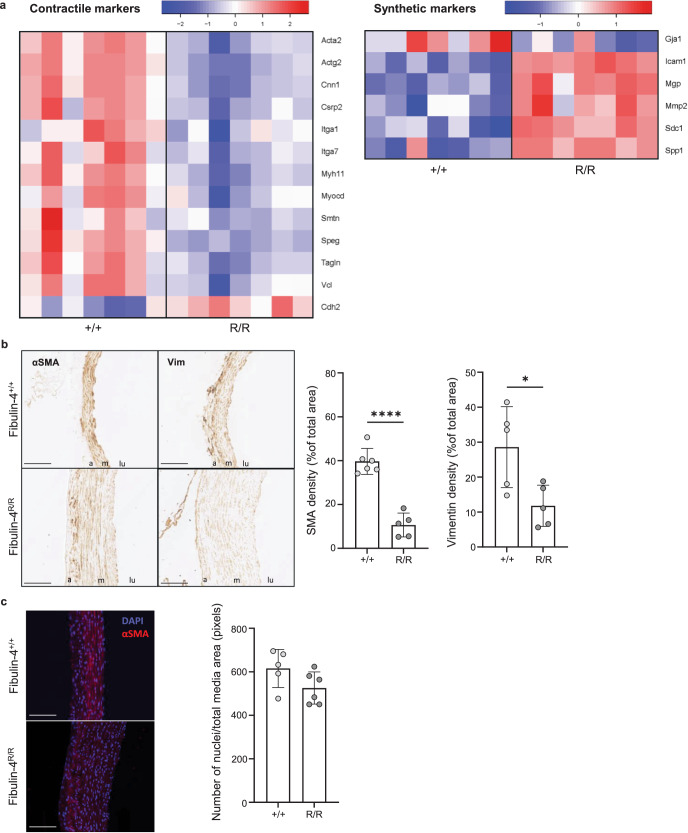
Phenotypic switching of VSMCs in the Fibulin-4 mutant mouse aorta. **a** Heatmap of significantly differentially expressed genes that are markers for the contractile VMSC phenotype and the synthetic VSMC phenotype (*p* ≤ 0.05). **b** Staining (left) and quantification (right) for contractile marker αSMA and synthetic marker vimentin on *Fibulin-4*^*R/R*^ and *Fibulin-4*^*+/+*^ mouse aortas. The mean percentage (±SD) of DAB staining of the total aortic media surface area is plotted (*n* = 5–6 per group, *p < 0.05, ****p < 0.0001, unpaired *t* test). Bar = 100 mm, m media, a adventitia, lu lumen. **c** Staining (left) and quantification (right) of DAPI staining on *Fibulin-4*^*R/R*^ and *Fibulin-4*^*+/+*^ mouse aortas to determine the number of nuclei, with an αSMA staining as reference for the aortic media. The mean number (±SD) of nuclei per total media area in pixels is plotted (*n* = 5–6 per group, *p* > 0.05, unpaired *t* test).

A phenotypic switch of VSMCs from a contractile to a synthetic phenotype often appears to be involved in aortic dysfunction^16^. Therefore, we investigated the expression of genes that are markers for either a contractile or a synthetic VSMC phenotype in our RNA expression data based on literature (Supplementary Table 3)^21^. Of the 17 investigated markers for a contractile phenotype, 12 were downregulated in the *Fibulin-4*^*R/R*^ aortic arch (Fig. 3a and Supplementary Fig. 2). The two most suitable marker proteins for a contractile phenotype are smoothelin (encoded by *Smtn*) and SM-MHC (encoded by *Myh11*) and both were significantly downregulated^21^. Of the 12 investigated markers for a synthetic phenotype, five were upregulated and one was downregulated (Fig. 3a and Supplementary Fig. 2). These findings suggested a phenotypic switch of VSMCs in the *Fibulin-4*^*R/R*^ aortic arch from a contractile to a synthetic phenotype.

To validate the RNAseq findings suggesting a less contractile, more synthetic VSMC phenotype in the *Fibulin-4*^*R/R*^ aortic arch, immunohistochemical staining was performed on mouse aorta for contractile marker αSMA and synthetic marker vimentin (Fig. 3b). Both αSMA and vimentin staining were significantly decreased in the *Fibulin-4*^*R/R*^ compared to the *Fibulin-4*^*+/+*^ aorta after correction for total media area. To assess whether this is caused by a loss of VSMCs in the medial layer rather than by phenotypic switching, a staining for DNA (DAPI) and a counterstain with αSMA, to indicate the aortic media, was performed (Fig. 3c). After correction for total aortic media area, no significant change was observed in the number of VSMCs in the *Fibulin-4*^*R/R*^ aortic arch compared to the *Fibulin-4*^*+/+*^ aortic arch. This suggested that the observed decreased expression of αSMA and vimentin in the *Fibulin-4*^*R/R*^ aortic arch was due to phenotypic changes of VSMCs, but did not necessarily support a switch from a contractile to a synthetic phenotype.

### Increased expression of SASP factors, senescence markers p21, p16, and senescence-associated β-galactosidase (SA-β-gal) in the *Fibulin-4*^*R/R*^ aortic arch

**Fig. 4:**
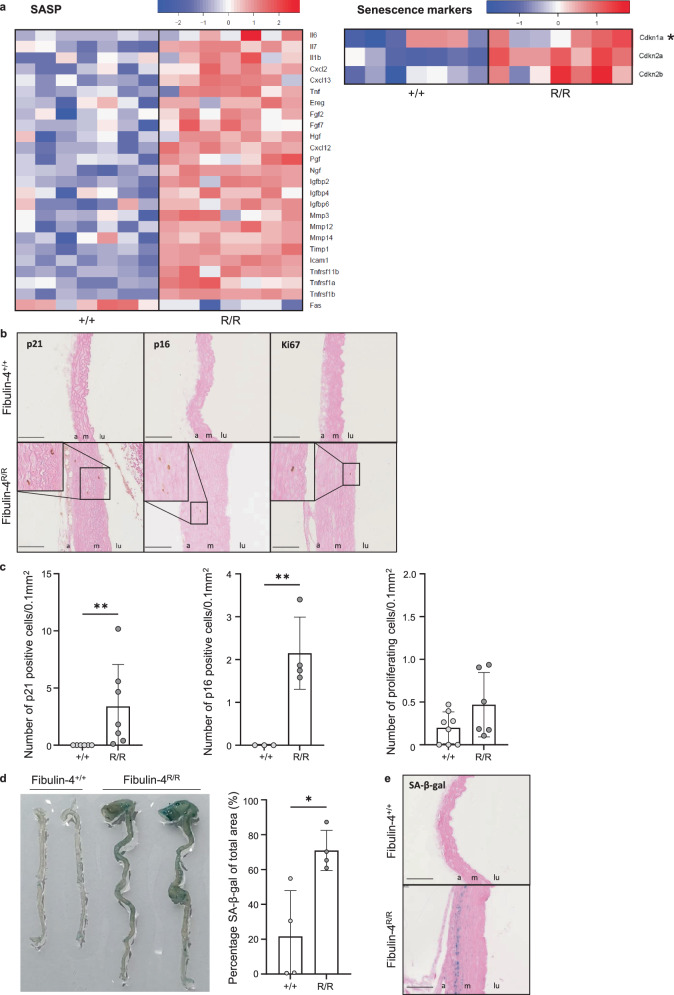
Differential expression of SASP factors and staining for senescence markers suggest senescence of cells in Fibulin-4 mutant mouse aorta. **a** Heatmap of significantly differentially expressed SASP factors and senescence markers p21 (Cdkn1a)*, p16 (Cdkn2a) and p15 (Cdkn2b) in the *Fibulin-4*^*R/R*^ aortic arch compared to *Fibulin-4*^*+/+*^controls (*p* ≤ 0.05). *Note: Cdkn1a was not significantly changed (*p* = 0.302), all other displayed genes were. **b** Immunohistochemical staining for senescence markers p21 (left), p16 (middle), and Ki67 (right) on *Fibulin-4*^*R/R*^ and *Fibulin-4*^*+/+*^ mouse aorta. Bar = 100 mm. **c** Quantification of staining for p21, p16, and Ki67 on *Fibulin-4*^*R/R*^ and *Fibulin-4*^*+/+*^ mouse aortas (for p21; *n* = 6–7 per group, **p < 0.01, unpaired *t* test, for p16; *n* = 3–4 per group, **p < 0.01, unpaired *t* test and for Ki67; *n* = 6–8 per group, *p* > 0.05, unpaired *t* test). The mean number (±SD) of positive cells per 0.1 mm^2^ aortic media surface area is plotted per mouse. **d** Staining (left) and quantification (right) of senescence-associated β-galactosidase (SA-β-gal) on *Fibulin-4*^*R/R*^ and *Fibulin-4*^*+/+*^ mouse aortas. The mean percentage (±SD) SA-β-gal of the total area is plotted (*n* = 4 per group, *p < 0.05, unpaired *t* test). **e** SA-β-gal staining on *Fibulin-4*^*R/R*^ and *Fibulin-4*^*+/+*^ mouse aortas showing localization of SA-β-gal positive cells. Bar = 100 mm, m media, a adventitia, lu lumen.

We further investigated the RNAseq data for an alternative explanation for the altered phenotypic state of VSMCs in the *Fibulin-4*^*R/R*^ aorta. Several indications exist for the involvement of senescence in aneurysm development. A main feature of senescent cells is the secretion of senescence-associated secretory phenotype (SASP) factors, including pro-inflammatory cytokines, chemokines, MMPs, and ECM proteins. Therefore, we investigated the expression of 71 (groups of) genes that belong to the SASP phenotype based on literature (Fig. 4a and Supplementary Table 4)^22^. Of the investigated SASP factors, 39.4% was significantly changed in the *Fibulin-4*^*R/R*^ aortic arch compared to the *Fibulin-4*^*+/+*^ aortic arch (Fig. 4a, Supplementary Fig. 3, and Supplementary Table 5). Most factors were upregulated, which suggested the presence of senescent cells secreting SASP factors. In the *Fibulin-4*^*R/R*^ aortic arch, expression of MMP3, MMP12, MMP14, and tissue inhibitor of metalloproteinase-1 (TIMP1) was upregulated, and 16 genes encoding for different types of collagen chains were differentially expressed (Figs. 2c and 4a). This indicated that MMP expression and ECM remodeling were increased, which has been previously demonstrated in the *Fibulin-4*^*R/R*^ aorta^8,23^. Furthermore, expression of inflammatory factors interleukin-1B (IL-1B), interleukin-6 (IL-6), interleukin-7 (IL-7), C-X-C motif chemokine ligand 3 (CXCL3) and 13 (CXCL13) was upregulated in the *Fibulin-4*^*R/R*^ aortic arch, of which the pro-inflammatory cytokine IL-6 is the most prominent cytokine of the SASP^22^. Additionally, expression of CDKN2A (p16^INK4A^) and CDKN2B (p15^INK4B^), both cyclin-dependent kinase inhibitors involved in establishing cell cycle arrest in senescence, was significantly increased in the *Fibulin-4*^*R/R*^ aorta. To confirm these RNAseq findings, qPCR was performed which confirmed significantly increased expression of *Igfbp2*, *Timp1*, *Mmp3*, *Fn1*, *Col8a1*, *Col2a1*, *Il6*, *Il1b*, *Cdkn1a*, *Cdkn2a*, and *Cdkn2b* in the *Fibulin-4*^*R/R*^ aorta (Supplementary Fig. 4). Altogether, these findings suggested the presence of senescent cells in the *Fibulin-4*^*R/R*^ aortic arch.

To validate findings from our RNAseq data, immunohistochemical staining for senescence markers p21 and p16 was performed on *Fibulin-4*^*R/R*^ and *Fibulin-4*^*+/+*^ aortas. A significant increase was observed in p21 and p16 positive cells in the *Fibulin-4*^*R/R*^ aortic arch (Fig. 4b, c). These p21 and p16 positive cells were mainly present in the media, suggesting senescence of VSMCs. Ki67 staining revealed some proliferating cells in the *Fibulin-4*^*R/R*^ aorta, however, there was no significant increase compared to *Fibulin-4*^*+/+*^ mice. Additionally, a SA-β-gal staining on mouse aorta revealed increased staining of this marker in the *Fibulin-4*^*R/R*^ aorta, mainly visible in VSMCs residing in the medial layer (Fig. 4d, e). Increased staining for senescence markers p21, p16, and SA-β-gal in the *Fibulin-4*^*R/R*^ aorta, as well as no increase in Ki67 staining, confirmed the findings from our RNAseq data and suggested senescence of *Fibulin-4*^*R/R*^ VSMCs. Although cells that were positive for most of these markers were also visible in the thoracic and abdominal part of the aorta, their presence was most pronounced in the aneurysmal aortic arch (Supplementary Fig. 5).

### Increased p21 luminescence in the *Fibulin-4*^*R/R*^ aortic arch

**Fig. 5:**
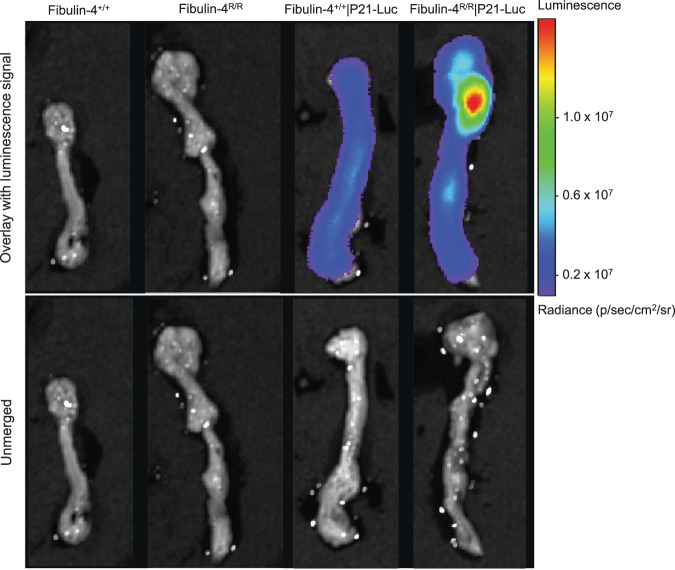
Increased p21 luminescence in Fibulin-4 mutant mouse aorta. Aortic luminescence of *Fibulin-4*^*+/+*^, *Fibulin-4*^*R/R*^, *Fibulin-4*^*+/+*^|p21-Luc and *Fibulin-4*^*R/R*^|p21-Luc mice (*n* = 2 per group). The top row shows the images merged with the luminescence signal and the bottom row shows the unmerged images (without the luminescence signal).

An important factor in the response to different stress stimuli, including DNA damage, that can trigger the induction of cellular senescence, is p21^24^. We generated *Fibulin-4*^*R/R*^|p21-Luciferase mice in which expression of firefly luciferase was placed under the control of the endogenous p21 promoter. *Fibulin-4*^*R/R*^|p21-Luciferase aortas showed increased luminescence compared to *Fibulin-4*^*+/+*^|p21-Luciferase aortas (Fig. 5). As a control, both *Fibulin-4*^*R/R*^ and *Fibulin-4*^*+/+*^ mice without p21 luciferase showed no luminescence. These results confirmed the presence of increased p21 expression in the aortic arch of *Fibulin-4*^*R/R*^|p21-Luciferase mice.

### *Fibulin-4*^*R/R*^ VMSCs exhibit increased senescence markers in vitro

**Fig. 6:**
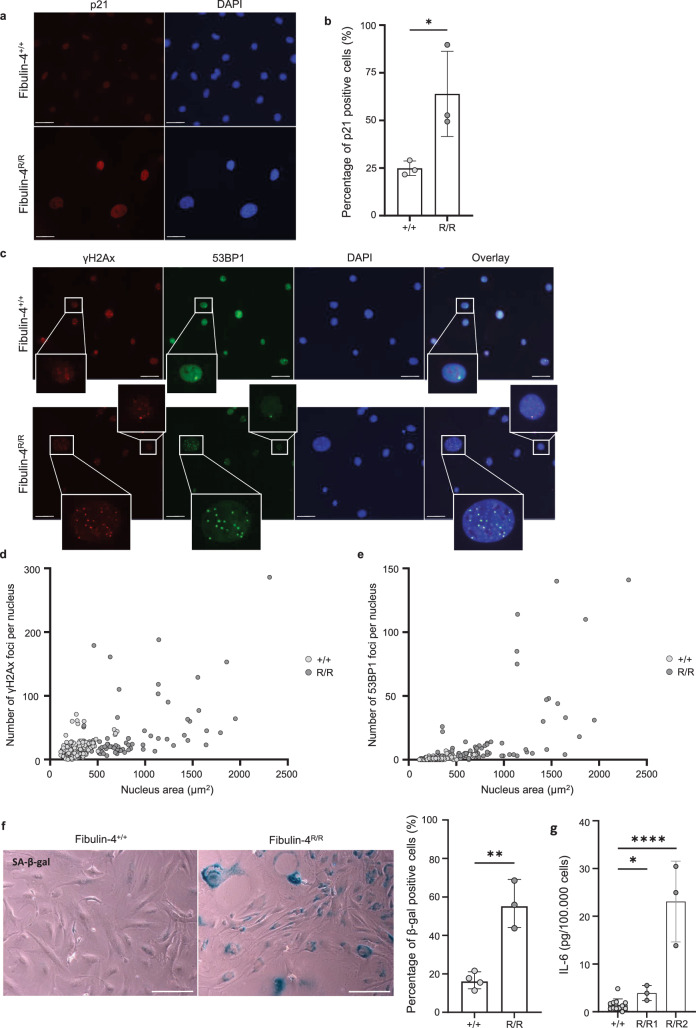
Staining for senescence markers suggests increased senescence of Fibulin-4 mutant mouse VSMCs in vitro*.* **a** Immunofluorescent staining for senescence marker p21 on *Fibulin-4*^*R/R*^ and *Fibulin-4*^*+/+*^ VSMCs. Bar = 50 µm. **b** Quantification of p21 positive cells in *Fibulin-4*^*R/R*^ VSMCs and *Fibulin-4*^*+/+*^ controls. The percentage (mean ± SD) of p21 positive cells is plotted per cell line (*n* = 3, *p < 0.05, unpaired *t* test). **c** Immunofluorescent staining for DNA damage markers γH2Ax and 53BP1 on *Fibulin-4*^*R/R*^ and *Fibulin-4*^*+/+*^ VSMCs. Bar = 50 µm. **d** Plot showing the correlation between the number of γH2Ax foci per nucleus and nucleus area (μm^2^). Each data point represents an individual nucleus (*n* = 2–3 cell lines per group, 37–81 nuclei analyzed per cell line). There is a significant correlation between foci number and nucleus area in the *Fibulin-4*^*R/R*^ VSMC group (*p* < 0.0001, Spearman nonparametric correlation test). **e** Plot showing the correlation between the number of 53BP1 foci per nucleus and nucleus area (μm^2^). Each data point represents an individual nucleus (*n* = 2–3 cell lines per group, 37–81 nuclei analyzed per cell line). There is a significant correlation between foci number and nucleus area in the *Fibulin-4*^*R/R*^ VSMC group (*p* < 0.0001, Spearman nonparametric correlation test). **f** Staining (left) and quantification (right) of SA-β-gal in Fibulin-4^R/R^ and *Fibulin-4*^*+/+*^ VSMCs. The percentage (mean ± SD) of SA-β-gal positive cells is plotted per cell line (*n* = 3–4 per group, **p < 0.01, unpaired *t* test). Bar = 500 µm. **g** Plot showing the level of IL-6 (mean ± SD) secreted into the medium by *Fibulin-4*^*+/+*^ controls (*n* = 4) and two separate *Fibulin-4*^R/R^ VSMC lines. Experiments were performed in triplo, separate replicates were plotted (*p < 0.05, ***p < 0.001, unpaired *t* test).

Additionally, the senescent phenotype of *Fibulin-4*^*R/R*^ VSMCs was investigated in vitro. After isolation from the mouse aorta, VSMC phenotype was confirmed by immunofluorescent staining for the contractile markers SM22 and αSMA and synthetic markers vimentin and collagen I (Supplementary Fig. 6). Next, the expression of several senescence markers was assessed. After 7 days in culture, immunofluorescent staining was performed for the stress and senescence marker p21 (Fig. 6a). The percentage of p21 positive cells was significantly increased in *Fibulin-4*^*R/R*^ VSMCs compared to *Fibulin-4*^*+/+*^ VSMCs (Fig. 6b). Since senescent cells show chronic accumulation of DNA damage repair proteins (senescence-associated DNA damage foci), staining for γH2Ax and 53BP1 was performed (Fig. 6c). A significant increase of both γH2Ax and 53BP1 foci was observed in *Fibulin-4*^*R/R*^ VSMCs compared to *Fibulin-4*^*+/+*^ VSMCs (Fig. 6c and Supplementary Fig. 7a). Additionally, the correlation between foci number and nuclear size was investigated in *Fibulin-4*^*R/R*^ VSMCs, since increased nuclear size is another characteristic of the senescent phenotype. The nucleus area of *Fibulin-4*^*R/R*^ VSMCs was significantly increased compared to *Fibulin-4*^*+/+*^ VSMCs (Fig. 6d, e and Supplementary Fig. 7b). Correlation analysis revealed a significant correlation between nucleus area and γH2Ax and 53BP1 foci number in *Fibulin-4*^*R/R*^ VSMCs (Fig. 6d, e). Furthermore, SA-β-gal staining revealed significantly increased SA-β-gal activity in *Fibulin-4*^*R/R*^ VSMCs compared to *Fibulin-4*^*+/+*^ VSMCs (Fig. 6f). Lastly, *Fibulin-4*^*R/R*^ VSMCs showed increased secretion of SASP factor IL-6 in the culture medium in vitro (Fig. 6g). Altogether, these markers indicated that *Fibulin-4*^*R/R*^ VSMCs also have a senescent phenotype in vitro. The *Fibulin-4*^*R/R*^ mouse model was generated through transcriptional interference by placing a TKneo-targeted construct in the downstream gene *Mus81. Mus81* is involved in the homologous recombination DNA repair pathway, therefore knockout of this gene could result in an increased presence of DNA damage foci and induction of cellular senescence due to impaired DNA repair capacities. To confirm that the senescent phenotype (and especially the accumulation of DNA damage repair proteins) was not due to the knockout of *Mus81*, staining for SA-β-gal, γH2Ax, and 53BP1 was performed on *Fibulin-4*^*SMKO*^ VSMCs (Supplementary Fig. 8). These VSMCs express no Fibulin-4 protein and do not harbor the *Mus81* mutation. Similar to *Fibulin-4*^*R/R*^ VSMCs, *Fibulin-4*^*SMKO*^ VSMCs showed increased SA-β-gal staining and an increased number of γH2Ax and 53BP1 foci compared to their wildtype controls, confirming that this was caused by aberrant Fibulin-4 expression.

### Treatment of *Fibulin-4*^*R/R*^ VMSCs with AG490, TGF-β nAb, or navitoclax reduces senescence markers in vitro

To investigate therapeutic compounds that could alleviate the *Fibulin-4*^*R/R*^ phenotype, an upstream regulator analysis was performed in IPA. AG490, an inhibitor of the JAK/STAT pathway, was predicted to be inhibited as an upstream regulator (activation *z* score = −4.253). Additionally, IPA analysis revealed upregulation of signaling through STATs resulting in increased expression of downstream IL-6 and SOCS genes (Supplementary Fig. 9). The JAK/STAT pathway is activated in senescent cells, and inhibition of this pathway suppresses the SASP^25^. These findings suggest that the JAK/STAT pathway could be a possible therapeutic target. Furthermore, TGFB1 and TGFB3, activators of the TGF-β pathway, were both predicted to be activated as upstream regulators (activation *z* score = 3.938 and 2.643, respectively). As previously mentioned, increased TGF-β signaling was observed in the aortic arch of *Fibulin-4*^*R/R*^ mice (Fig. 1d, e). Additionally, a previous study revealed that treatment of *Fibulin-4*^*R/R*^ VSMCs with TGF-β neutralizing antibodies (nAb) reversed their reduced growth, suggesting that this could also alleviate the senescent VSMC phenotype^11^. Lastly, different senolytic compounds including quercetin, fisetin, curcumin, resveratrol, and metformin were predicted to be inhibited as upstream regulators (activation *z* score = −2.103, −2.051, −4.168, −3.039, and −2.897, respectively)^26^. Together with the observation of increased expression of senescence markers in *Fibulin-4*^*R/R*^ VSMCs, these findings suggest the therapeutic potential of senolytics. In conclusion, upstream regulator analysis uncovered multiple possible therapeutic targets.

**Fig. 7:**
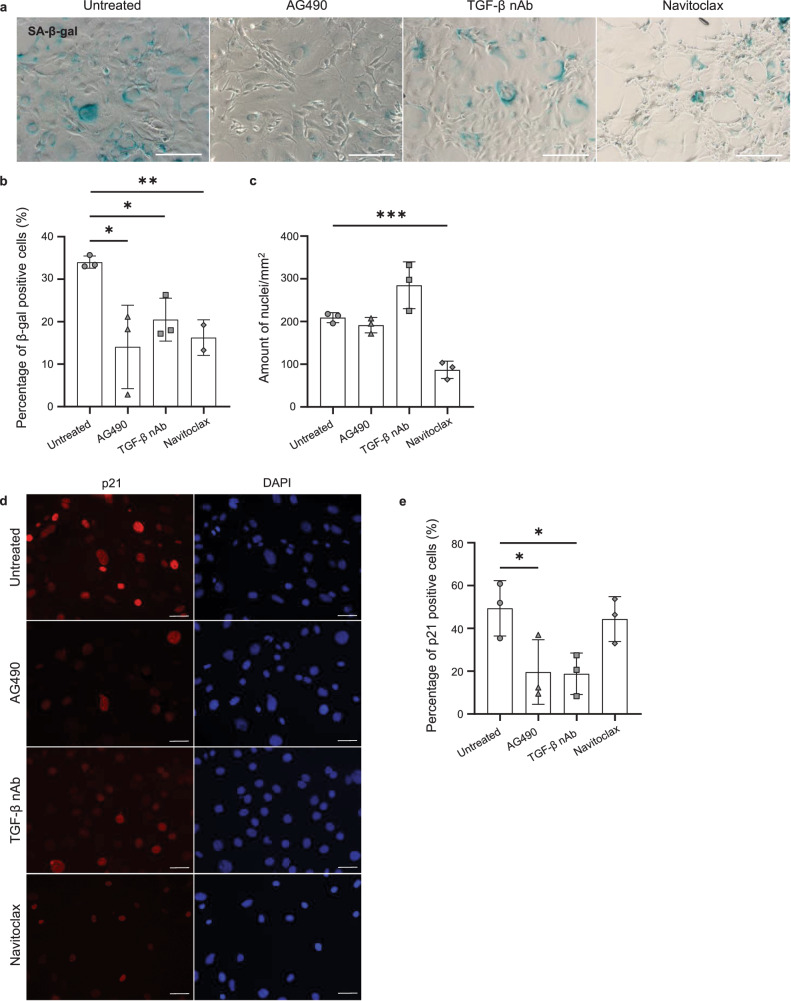
Treatment of Fibulin-4 mutant mouse VSMCs with AG490, TGF-β nAb, and navitoclax reduces senescence markers in vitro*.* **a** SA-β-gal staining on *Fibulin-4*^*R/R*^ VSMCs treated with AG490, TGF-β nAb, or navitoclax for 48 h (or untreated). Bar = 500 µm. **b** Quantification of SA-β-gal staining on *Fibulin-4*^*R/R*^ VSMCs. The percentage (mean ± SD) of SA-β-gal positive cells is plotted (*p < 0.05, **p < 0.01, unpaired *t* test). **c** Quantification of the amount of nuclei per mm^2^ (mean ± SD, ***p < 0.001, unpaired *t* test). **d** Immunofluorescent staining for p21 on *Fibulin-4*^*R/R*^ VSMCs treated with AG490, TGF-β nAb, or navitoclax for 48 h (or untreated). Bar = 50 µm. **e** Quantification of the percentage (mean ± SD) of p21 positive cells (*p < 0.05, unpaired *t* test). All experiments were performed in triplo, separate replicates were plotted. Note: SA-β-gal analysis after navitoclax treatment was performed in duplo.

To investigate whether possible therapeutic compounds could reduce senescence in vitro, *Fibulin-4*^*R/R*^ VSMCs were treated with AG490, TGF-β nAb or navitoclax. AG490 treatment significantly reduced the percentage of SA-β-gal positive cells and the percentage of p21 positive cells in *Fibulin-4*^*R/R*^ VSMCs in vitro (Fig. 7a, b, d, and e). Furthermore, the therapeutic effect of TGF-β nAb was assessed in vitro. TGF-β nAb treatment significantly reduced the percentage of SA-β-gal positive cells and the percentage of p21 positive cells in *Fibulin-4*^*R/R*^ VSMCs in vitro (Fig. 7a, b, d, and e). Additionally, although not significant (*p* = 0.0796, unpaired *t* test), a trend of increased cell number was observed after treatment, suggesting increased proliferation of VSMCs (Fig. 7c). Lastly, the therapeutic effect of the senolytic compound navitoclax was assessed. Navitoclax treatment significantly reduced the percentage of SA-β-gal positive cells, as well as the total cell number in *Fibulin-4*^*R/R*^ VSMCs in vitro (Fig. 7a–c). However, navitoclax treatment did not affect the percentage of p21 positive cells, suggesting that the remaining VSMCs stay in cell cycle arrest (Fig. 7d, e). Altogether, these results demonstrated the therapeutic effect of AG490, TGF-β nAb, and navitoclax on *Fibulin-4*^*R/R*^ VSMCs in vitro and indicated the involvement of the JAK/STAT and TGF-β signaling pathway in establishing the senescent phenotype.

## Discussion

Aortic aneurysms are a life-threatening condition that can be fatal without timely surgical repair. Up to now, pharmacological treatment is lacking, mostly because there are still gaps of knowledge regarding the molecular mechanism underlying aneurysm formation. Mutations in the *Fibulin-4* gene result in the development of cutis laxa syndrome in human patients, characterized by aortic aneurysm formation. To investigate the underlying molecular mechanisms of aneurysm disease development, this deficiency was mimicked in *Fibulin-4*^*R/R*^ mice. Previous research showed alterations in the TGF-β signaling pathway, ECM remodeling, aberrations of the cytoskeleton, and mitochondrial dysfunction in this mouse model^8–10,12^. We aimed to further identify the effect of *Fibulin-4* mutation in the mouse aorta at the molecular level to explain the underlying mechanism of aneurysm formation using an RNA sequencing approach.

Several findings from our RNA expression analysis confirm data presented in previous research on *Fibulin-4*^*R/R*^ mice. Similar to previous findings, our data shows that downstream TGF-β signaling in the *Fibulin-4*^*R/R*^ aortic arch is increased. Additionally, previous proteomics and microarray analyses of 3-month-old *Fibulin-4*^*R/R*^ mouse aorta revealed significant changes in pathways related to mitochondrial function and metabolism^9^. We compared our RNA sequencing results with these previously published microarray data. Although our current study was different in platform, reference analysis, and amount of significantly differentially expressed molecules, the identified shared pathways included metabolic pathways, PPAR, RAS, and MAPK/ERK signaling. Additionally, a comparison analysis of the top 20 inhibited and top 20 activated upstream regulators predicted to be significantly regulated in both datasets showed an overlap of 20 molecules, including *Pparg* and *Il1b*. Furthermore, IPA analysis reveals a change in genes and pathways involved in the cytoskeleton and ECM remodeling in *Fibulin-4*^*R/R*^ mice. Accumulation and disorganization of elastin fibers and accumulation of proteoglycans in the medial layer of the *Fibulin-4*^*R/R*^ aortic arch confirm these findings. This shows that analysis of the current RNAseq data properly uncovered important processes at play in the *Fibulin-4*^*R/R*^ aneurysmal aorta, supporting the fact that the other pathways and molecules identified are equally valid.

Analysis of the *Fibulin-4*^*R/R*^ aortic media shows increased medial wall thickness, but no significant change in the number of VSMCs (after correction for surface area) in the *Fibulin-4*^*R/R*^ aortic arch compared to the *Fibulin-4*^*+/+*^ aortic arch. Additionally, Ki67 staining reveals some proliferating cells in the *Fibulin-4*^*R/R*^ aorta, but no significant increase compared to *Fibulin-4*^*+/+*^ mice. As the proliferation of VSMCs in the aortic media of 10-day-old *Fibulin-4*^*R/R*^ mice is increased, it is possible that VSMCs contribute to the observed increased wall thickness in an earlier stage of aneurysm growth, but may be less involved in later stages^8^.

VSMCs show high phenotypic plasticity and can switch from a functional, contractile phenotype to a proliferative, synthetic phenotype upon stress signals to enable vascular remodeling. Phenotypic switching of VSMCs is thought to play a role early in aneurysm formation, causing pathological remodeling of the vessel wall^16,27^. IPA analysis reveals the downregulation of markers for the contractile VSMC phenotype and upregulation of synthetic markers in the *Fibulin-4*^*R/R*^ aortic arch. Interestingly, *Fibulin-4*^*R/R*^ aortas show decreased expression of both contractile marker αSMA and synthetic marker vimentin. Previous research showed increased αSMA and SM22 protein levels in *Fibulin-4*^*R/R*^ VSMCs in vitro, which would indicate a more contractile phenotype^10^. However, *Fibulin-4*^*R/R*^ VSMCs exhibit aberrant cytoskeletal structures and ECM production, which impair contractility, still causing an aberrant contractile phenotype. As previously mentioned, downstream signaling in the TGF-β pathway is increased in *Fibulin-4*^*R/R*^ mice^28^. TGF-β signaling is known to regulate the differentiation of VSMCs by targeting VSMC markers such as αSMA, SM22 and calponin 1, and abnormal TGF-β signaling could therefore be responsible for the observed phenotypic changes^29,30^. Even though the aortic staining for vimentin does not indicate a switch to a synthetic phenotype, the RNAseq data shows significant upregulation of several other synthetic markers. This suggests that *Fibulin-4*^*R/R*^ VSMCs undergo a phenotypical change that might be involved in aneurysm formation, such as the adaptation of a senescent phenotype, in which VSMCs change from the contractile to the synthetic phenotype^31,32^.

Differential expression data suggests the presence of senescent cells in the *Fibulin-4*^*R/R*^ aortic arch. The presence of senescent cells in the *Fibulin-4*^*R/R*^ aortic arch is confirmed ex vivo in aortic tissue and in vitro in VSMCs, by investigation of senescence markers p21, p16, SA-β-gal, DNA damage foci, and SASP factor IL-6. Senescence of VSMCs has been previously observed in several cardiovascular diseases, amongst which aortic aneurysms^33–36^. We now further provide evidence that suggests the involvement of VSMC senescence in aortic aneurysm development. TGF-β can induce the expression of cell-cycle inhibitors p21, p16^INK4A^, and p15^INK4B^ and thereby induce senescence^37,38^. A previous study already showed reduced proliferation of *Fibulin-4*^*R/R*^ VSMCs in vitro, indicating cell cycle arrest which is also characteristic of the senescent phenotype^11,39^. Additionally, TGF-β was shown to induce the production of reactive oxygen species (ROS) in the mitochondria of several cell types^40^. ROS can induce DNA damage which eventually can trigger senescence^41–44^. Mitochondrial dysfunction and increased ROS production were previously observed in *Fibulin-4*^*R/R*^ VSMCs, and we now also observe increased DNA damage in these cells^9^. Furthermore, previous research showed increased senescence of VSMCs from patients with Marfan syndrome^45^. Much like in patients and mice with *Fibulin-4* mutations, TGF-β signaling is increased in Marfan syndrome and it is thought that TGF-β might trigger the observed senescence of these VSMCs^33^. Our results show that inhibition of the TGF-β pathway using TGF-β neutralizing antibodies reduces senescence markers SA-β-gal and p21 in *Fibulin-4*^*R/R*^ VSMCs in vitro. Therefore, our findings further evidence the involvement of the TGF-β pathway in establishing the senescence phenotype and indicate that targeting of the TGF-β pathway is effective in alleviating the senescent phenotype.

Another proposed mechanism of senescence induction is mechanical stress. The structure of the vessel wall of *Fibulin-4*^*R/R*^ mice is already compromised, making it less resistant to mechanical strain caused by cyclic pulsation of the blood^46,47^. Furthermore, stiffening of the aorta reduces arterial compliance, causing pulse pressure (the difference between systolic and diastolic blood pressure) to increase^48^. Indeed, our RNAseq data analysis reveals that mechanosensing is disturbed in the *Fibulin-4*^*R/R*^ aortic arch. The ECM is important in the transduction of mechanical stimuli from the surrounding environment and in the *Fibulin-4*^*R/R*^ aorta we observe an aberrant ECM, which could explain why mechanosensing is disturbed. Abnormal or excessive mechanical stimuli can induce pathological phenotypical changes in VSMCs, and therefore possibly also trigger senescence. Additionally, compositional changes of the ECM can trigger phenotypic modulation of VSMCs, also contributing to this process^49^.

VSMC senescence has been previously associated with thoracic aortic aneurysm formation in patients^33,50^. In mice, senescence of VSMCs has only been observed in models for abdominal aortic aneurysms^51,52^. Senescence of VSMCs is associated with persistent DNA damage, as we also observe, and the presence of a SASP induces degradation of the ECM, as well as triggers an inflammatory response^22^. Both ECM degradation and inflammation impair vessel wall structure and function, further promoting aneurysm formation^53^. Furthermore, the SASP includes secreted molecules, amongst which TGF-β, that can induce paracrine senescence and maintain the senescent phenotype^40^. Altogether, the senescence of VSMCs in the aortic wall potentially contributes to aortic aneurysm formation.

The JAK/STAT signaling pathway is activated in senescent cells and inhibition of this pathway has been shown to suppress SASP secretion and attenuate senescence^25,54^. Additionally, JAK/STAT signaling contributes to the maintenance of the cytokine profile in aneurysmal aorta^55^. Our results show that treatment of *Fibulin-4*^*R/R*^ VSMCs with the JAK/STAT inhibitor AG490 reduces senescence markers SA-β-gal and p21 in vitro. This further evidences that the JAK/STAT pathway plays a role in the induction of the senescent phenotype.

Treatment with the senolytic compound navitoclax reduces senescence marker SA-β-gal in *Fibulin-4*^*R/R*^ VSMCs in vitro. These results indicate that it would be of interest to specifically target senescent cells as a form of therapy. Treatment with senolytic agents dasatinib and quercetin already showed effectivity against abdominal aortic aneurysm growth in aged mice infused with angiotensin II, where reduced expression of Mmp2 and Mmp9 was observed upon treatment^56^. Additionally, navitoclax treatment of Ang II-induced premature senescent VSMCs in vitro also reduced the expression of Mmp2 and Mmp9. These studies indicate that senescence could be targeted to prevent or reduce aneurysm development^57^. Importantly, specifically targeting senescent cells as a form of therapy also shows potential for translation to the clinic, as senescence of VSMCs has been identified in the aortas of patients with Marfan syndrome. However, it should be noted that there are also risks to using senolytic treatment. Apoptosis of a large number of senescent VSMCs in the aneurysmal aorta could promote wall instability if they are not directly replaced by healthy VSMCs. Therefore, the use of senomorphics, compounds that block or regulate the secretion of SASP factors, could also be considered^58^. Lastly, since targeting the JAK/STAT and TGF-β pathway reduces senescence markers in vitro, the treatment that reduces JAK/STAT and TGF-β pathway activity could also be therapeutic in preventing the senescent phenotype.

In conclusion, these results further reveal underlying molecular mechanisms of aortic aneurysm development in *Fibulin-4*^*R/R*^ mice. Our studies demonstrate that *Fibulin-4*^*R/R*^ VSMCs show a senescent phenotype which might contribute to impaired functioning of the aortic wall. Furthermore, our results indicate the involvement of the JAK/STAT and TGF-β pathway in establishing this senescent phenotype. Notably, the *Fibulin-4*^*R/R*^ mouse model is representative of thoracic aneurysm formation in patients and is a suitable model to investigate its underlying mechanisms. These findings form interesting leads for further investigation and can contribute to improved therapeutic options to alleviate or even prevent aortic aneurysm formation. Importantly, by generating *Fibulin-4*^*R/R*^|p21-Luciferase mice, we can now study the effect of therapeutic treatment on both senescence and aneurysm development simultaneously, as well as longitudinally, providing a tool to study potential new anti-senescence treatment options.

## Methods

### Experimental animals

*Fibulin-4*^*+/R*^ mice were bred into a C57BL6 background to obtain *Fibulin-4*^*R/R*^ and wild-type (*Fibulin-4*^*+/+*^) experimental animals. *Fibulin-4*^*SMKO*^ animals, with a VSMC-specific deletion of Fibulin-4, were kindly provided by Hiromi Yanagisawa and bred into the same C57BL6 background. Mice were fed a normal chow diet. Animals were housed at the Animal Resource Centre (Erasmus University Medical Centre), which operates in compliance with the “Animal Welfare Act” of the Dutch government, using the “Guide for the Care and Use of Laboratory Animals” as its standard. As required by Dutch law, formal permission to generate and use genetically modified animals was obtained from the responsible local and national authorities. An independent Animal Ethics Committee consulted by Erasmus Medical Center (CCD) approved these studies (permit number AVD1010020186886), in accordance with national and international guidelines. For the described experiments mice were sacrificed by CO_2_ inhalation.

### RNA sequencing

*Fibulin-4*^*R/R*^ and *Fibulin-4*^*+/+*^ littermate control mice were sacrificed at the age of 3 months (*n* = 7 per group, 3 male and 4 female). Total RNA and µRNA were obtained from the aortic arch and abdominal aortas using the miRNeasy Mini Kit (Qiagen). Library preparation, sequencing, and primary data analysis were performed at GenomeScan B.V. (Genomescan B.V., Leiden, The Netherlands). Libraries were single-end sequenced in an Illumina NextSeq500 platform at a sequencing depth of 12 million reads and 75 bp read length. Reads were mapped to the reference sequence Mus_Musculus_GRCm38.p3 using a short read aligner based on Burrows-Wheeler Transform with a default matching rate of 2%. Read counts were generated using htseq-count. Differential expression analysis was performed with DESeq2 v1.10.1, a statistical package within the R platform v2.15.3, to compare gene expression in the aortic arch and abdominal aorta of mutant mice to gene expression in control mice with an adjusted *p* value threshold of 0.05^59,60^. The sequencing data were uploaded to the Galaxy web platform^61^. The Volcano Plot tool was used to create volcano plots, in which the top 20 most upregulated genes and the top 20 most downregulated genes were highlighted (ranked based on fold change). The heatmap2 tool was used to create heatmaps of pathways and processes of interest. The expression of each gene was scaled individually based on the rlog normalized counts.

### IPA

For the comparison of *Fibulin-4*^*R/R*^ with *Fibulin-4*^*+/+*^ sample groups (aortic arch and abdominal aorta), normalized expression values of 22,779 genes were uploaded into IPA (IPA^©^ software version 107193442, QIAGEN, Redwood City, California, USA). An IPA core analysis was performed on significantly differentially expressed genes with a fold change of −1.2 ≤ FC ≥ 1.2 and a *p* value ≤ 0.05, of which the numbers are indicated per sample group in Supplementary Table 1. The top 20 up- and downregulated genes, canonical pathways, and upstream regulators were investigated (see Supplementary Fig. 10 for an analysis overview). The top up- and downregulated genes were identified by ranking based on fold change. The top canonical pathways were ranked based on significance (*p* value ≤ 0.05, −log (*p* value) ≥ 1.3). A *z* score ≥ 2 indicates prediction of activation of the pathway and a *z* score ≤ −2.0 indicates prediction of inhibition of the pathway. Upstream regulators were selected based on *z* score significance cut-offs set to −2.0 ≤ *z* score ≥ 2.0 and *p* value ≤ 0.05. For the targeted analysis, canonical pathways of interest were searched manually in IPA. For the TGF-β and JAK/STAT pathway, an overlay of the dataset on the pathway was created to visualize which genes were significantly changed. For analysis of contractile and synthetic VSMC markers and SASP factors, separate gene lists were manually created in IPA based on literature (Supplementary Tables 3 and 4, respectively)^21,22^.

### RT-qPCR

Total RNA was reverse transcribed into cDNA using the iScript cDNA Synthesis Kit (#1708890, Bio-Rad) and by running the following program on the Arktik Thermal Cycler (Thermo Scientific): 1) 5 min at 25 °C, 2) 30 min at 42 ^o^C and 3) 5 min at 85 °C. Real-time qPCR was performed on 5 ng cDNA per sample using the iQ SYBR Green Supermix (#1708886, Bio-Rad) and by running the following program on the Bio-Rad CFX384 real-time system: (1) 3 min at 95 °C, 2) 40 cycles of 15 s at 95 °C and 30 s at 55 or 60 °C (depending on primers) and 3) 5 s at 55 or 60 °C. Primer sequences are listed in Supplementary Table 6. Each sample was run in duplicate and samples with duplicates deviating > 6% were excluded from the analysis. Genes were normalized to their respective housekeeping gene; *Hprt* for genes with 55 °C primers and *Ppia* for genes with 60 °C primers. The relative expression of genes was compared between *Fibulin-4*^*R/R*^ and *Fibulin-4*^*+/+*^ samples.

### Immunohistochemical staining of mouse aortic tissue

For histological analysis, *Fibulin-4*^*+/+*^ (*n* = 11, 8 male and 3 female) and *Fibulin-4*^*R/R*^ (*n* = 9, 4 male and 5 female) mice were sacrificed at an age of 3–4 months. Mice were perfused through the left ventricle with phosphate-buffered saline (PBS). Aortas were fixed in formalin, dehydrated through the histokinette processor (Microm), and subsequently paraffin-embedded, after which 4 µm sections were prepared and placed on slides. Hematoxylin and eosin (HE) staining was performed to assess general pathology. Media and lumen size were quantified by manual selection of the media and lumen using the freehand region tool in NDP view.2 (Hamamatsu Photonics K.K., U12388-01), which calculates the surface area in mm^2^ (Supplementary Fig. 11a). A Movat’s pentachrome staining was performed for simultaneous visualization of different components of the vessel wall^62^, Resorcin-Fuchsin (RF) staining was performed to visualize the elastin structure and Alcian blue (AB) staining was performed to assess the ECM (stain for proteoglycans). AB staining was quantified using Fiji (ImageJ)^63^ (Supplementary Fig. 11b).

For immunohistochemical analysis, aorta sections were first deparaffinized and boiled in antigen retrieval buffer (10 mM Tris base, 1 mM EDTA solution, pH 9.0) at 300 W for 15 min. Slides were incubated in 3% H_2_O_2_ in methanol for 10 min to block endogenous peroxidase activity and subsequently blocked with 5% Protifar in PBS with 0.025% Triton X-100 for 1 h. Slides were incubated overnight at 4 °C with the following primary antibodies diluted in 1% Protifar in PBS with 0.025% Triton X-100; anti-αSMA (1:500 mouse monoclonal, ab7818, Abcam), anti-vimentin (1:500, ab2547, Abcam), anti-p21 (1:100 rat monoclonal [Hugo291], ab107099, Abcam), anti-p16 (1:200 rabbit monoclonal, ab211542, Abcam), and anti-Ki67 (1:200 rat monoclonal, Clone TEC-3, DAKO). The following day, slides were incubated with biotinylated secondary antibody (1:200, DAKO) for 30 min and subsequently with avidin-biotinylated complex (Vectastain Universal Elite ABC kit Vector Laboratories) for 30 min. DAB chromogen (DAKO Liquid Dab substrate-chromogen system) was used as a substrate and slides were counterstained with Eosin. Quantification of p21, p16, and Ki67 staining was performed by manually counting the number of positive cells and correcting for media size (Supplementary Fig. 11c). Quantification of αSMA and vimentin staining was performed using Fiji (ImageJ)^63^ (Supplementary Fig. 11d).

### Immunofluorescent staining of mouse aortic tissue

For immunofluorescent analysis, aorta sections were first deparaffinized and boiled in antigen retrieval buffer (10 mM Sodium Citrate, 0.05% Tween-20 solution, pH 6.0) at 300 W for 15 min. Tissue was permeabilized in TBS with 0.05% Triton X-100 for 10 min. Slides were blocked with 5% normal goat serum (NGS) + 0.3 M glycine in TBS with 0.05% Tween-20 for 1 h. Slides were incubated overnight at 4 °C with primary antibody (anti-αSMA (1:500 mouse monoclonal, ab7818, Abcam)) diluted in 1% NGS in TBS with 0.05% Tween-20. The following day, slides were incubated for 1 h at room temperature with a secondary antibody (anti-mouse Alexa 594, 1:1000) diluted in 1% NGS in TBS with 0.05% Tween-20. Slides were mounted with Vectashield containing DAPI (H-1200, Vector laboratories) and sealed with nail polish. Images were recorded on a near-infrared wide-field microscope (Axio Imager D2, Zeiss) using a 10× objective lens. Quantification of the number of nuclei in αSMA-positive area was performed using Fiji (ImageJ)^63^.

### Isolation of VSMCs from mouse aorta

To investigate VSMCs derived from the mutant mouse aorta in vitro, VSMCs were isolated from the aorta of *Fibulin-4*^*R/R*^ (*n* = 3), *Fibulin-4*^*+/+*^ (*n* = 4), *Fibulin-4*^*SMKO*^ (*n* = 3), and *Fibulin-4*^*SMWT*^ (*n* = 2) mice according to the protocol in Supplementary Table 7. In short, mice were sacrificed and perfused through the left ventricle with PBS. Aortas were isolated and incubated in 2 mg/ml collagenase type II (LS004176, Worthington) at 37 °C, 5% CO_2_ for 1–6 h until the tissue was dissolved. Primary VSMCs were centrifuged, and the cell pellet was resuspended in Dulbecco’s Modified Eagle’s Medium (DMEM) (Gibco) supplemented with 10% fetal calf serum (FCS) and 1% penicillin-streptomycin (PS) and cells were cultured on gelatinized dishes (0.1% gelatin) at 37 °C, 5% CO_2_.

### Immunofluorescent staining of VSMCs

To investigate phenotype and senescence markers, VSMCs were seeded on 0.1% gelatin-coated coverslips. VSMCs were incubated in culture for 7 days to allow the formation of ECM, and to ensure a better representation of in vivo conditions. After 7 days, VSMCs were fixed with 2% paraformaldehyde in PBS for 15 min. Fixated cells were washed with PBS + 0.1% Triton X-100 and blocked with PBS+ (PBS supplemented with 0.5% bovine serum albumin and 0.15% glycine) for 20 min. Coverslips were incubated overnight at 4 °C with the primary antibodies diluted in PBS+ as mentioned in Supplementary Table 8. Coverslips were washed with PBS + 0.1% Triton X-100 and shortly washed with PBS+ before incubation with the corresponding secondary antibodies diluted in PBS+ for 1 h at room temperature (see Supplementary Table 8). Additionally, SiR-Actin (1:1000, SC001, Tebu Bio) was added to visualize the cytoskeleton. After incubation, the coverslips were washed with PBS + 0.1% Triton X-100 and shortly washed with PBS, after which they were mounted on glass slides with Vectashield containing DAPI (H-1200, Vector laboratories) and sealed with nail polish. Images were recorded on a near-infrared wide-field microscope (Axio Imager D2, Zeiss) using a 20× objective lens. Images for quantification of the staining for 53BP1 and γH2AX were recorded on a TCS SP5 confocal microscope (Leica) using a 40× objective lens. The brightness and/or contrast of representative images shown in Figs. 6a, c and 7d was adjusted. This was performed equally for all images of each individual staining. Quantifications were performed on raw, unedited images using Fiji (ImageJ)^63^.

### SA-β-galactosidase (SA-β-gal) staining of VSMCs

To investigate SA-β-gal activity, VSMCs were seeded on 0.1% gelatin-coated coverslips. After 7 days, VSMCs were fixed and stained for SA-β-gal activity using the Senescence β-Galactosidase Staining Kit (#9860, Cell Signaling Technology) according to the protocol provided by the manufacturer. In short, VSMCs were fixed with 1× Fixative solution for 15 min at room temperature, after which they were washed with PBS and incubated with the β-galactosidase staining solution at 37 °C overnight in a dry incubator. Without removing the staining solution, images were recorded with an Olympus IX70 microscope using a Digital Color Camera (DFC300 FX, Leica). After washing with PBS, coverslips were mounted on glass slides with Vectashield containing DAPI (H-1200, vector laboratories) and sealed with nail polish. Images for quantification were recorded with a Leica DM4000 B microscope using a Digital Color Camera (DFC300 FX, Leica). Quantification was performed using Fiji (ImageJ)^63^ by counting the number of SA-β-gal positive cells in the bright-field channel and counting the total number of nuclei in the DAPI channel.

### Elisa

*Fibulin-4*^*R/R*^ and *Fibulin-4*^*+/+*^ VSMCs (*n* = 2–4) were seeded on 0.1% gelatin-coated 6 cm dishes. After 7 days, VSMCs were put on starvation medium (DMEM with 1% PS, without FBS). After 24 h, the medium was harvested and cells were counted. A sandwich ELISA was performed with the starvation medium to measure IL-6 secretion by VSMCs using the Mouse IL-6 Quantikine ELISA kit (M6000B, R&D Systems). Cell counts were used to correct the IL-6 levels to pg/100,000 cells. Experiments were performed in triplo.

### Treatment of VSMCs

*Fibulin-4*^*R/R*^ VSMCs were seeded in 12-well plates on 0.1% gelatin-coated coverslips. The medium was refreshed after 4 days. After 7 days, cells were treated with either 15 µg/ml TGF-β neutralizing antibodies (TGF-β 1,2,3 monoclonal antibody, MAB1835, R&D Systems), 25 µM AG-490 (S1143, Selleckchem) or 5 µM navitoclax (ABT-263, HY-10087, MedChemExpress) for 48 h. Immunofluorescent staining for p21 and SA-β-gal staining was performed according to the previously mentioned methods. Experiments were performed in triplo.

### P21 reporter assay

In order to evaluate p21 expression levels in vivo in mice, *Fibulin-4* mice were crossed with p21-luciferase reporter mice. The reporter construct was directed to the end of exon 3 of the endogenous p21 locus where a sequence, which encodes for T2A-β-gal-T2A-luciferase, was inserted^64^. This insertion led expression of p21, β-gal, and luciferase from the one engineered allele. *Fibulin-4*^*+/+*^ and *Fibulin-4*^*R/R*^ mice were crossed with p21-luciferase reporter mice, which led to both hetero- and homozygous animals for the reporter loci. Next, 3-month-old animals that were homozygous for the p21 reporter loci were selected (*n* = 2 for both *Fibulin-4*^*+/+*^|p21-Luciferase and *Fibulin-4*^*R/R*^|p21-Luciferase mice). First, mice were imaged for background levels. Then mice were injected with 150 mg/kg luciferin intraperitoneal and p21-luciferin activity was imaged 10–20 min after injection using the IVIS^®^ spectrum in vivo imaging system (Perkin Elmer). As a control for background luminescence, we used *Fibulin-4*^*+/+*^ and *Fibulin-4*^*R/R*^ mice without any reporter loci, which showed no bioluminescent signal upon injection of luciferin.

### SA-β-galactosidase staining of mouse aorta

To investigate SA-β-gal activity in aortas ex vivo, *Fibulin-4*^*R/R*^ (*n* = 4) and *Fibulin-4*^*+/+*^ (*n* = 4) mice were sacrificed and subsequently perfused through the left ventricle with PBS. Aortas were removed and placed in fixative buffer (1% formaldehyde, 0.1% glutaraldehyde, 2 mM MgCl_2_, 5 mM EGTA, 0.1 M sodium phosphate buffer pH 7.8) overnight at 4 °C. Aortas were washed (2 mM MgCl_2_, 0.01% sodium deoxycholate, 0.02% NP-40, 0.1 M sodium phosphate buffer pH 7.8) and stained with X-gal staining solution (5 mM potassium ferricyanide, 5 mM potassium ferrocyanide, 2% X-gal in DMF) overnight at 37 °C.

### Statistics

Statistical analysis is embedded in the core analysis of IPA. The *p* value is calculated using a right-tailed Fisher’s exact test and reflects the probability that the overlap of significant differentially expressed genes in the dataset with a certain process or pathway is due to random chance. The *z* score is calculated to provide predictions on processes that are upstream or downstream of differentially expressed genes.

Statistical analysis of the quantification of lumen and media size, AB staining, and immunohistochemical staining on aorta sections, as well as immunofluorescent staining and SA-β-gal staining on VSMCs was performed in GraphPad Prism 9.3.0 (GraphPad Software Inc., La Jolla, California, USA). The Shapiro–Wilk test was used to test for normal distribution of the data. An unpaired two-sided *t* test was performed to determine significant differences between two groups. For not normally distributed data, a Mann–Whitney test was performed to determine significance. For the calculation of the correlation between two parameters, the Spearman nonparametric correlation test was performed. For each statistical test, a *p* value < 0.05 was considered significant. All results are expressed as mean ± standard deviation (SD). In figures, one asterisk (*) indicates a *p* value < 0.05, two asterisks (**) indicate a *p* value < 0.01, three asterisks (***) indicate a *p* value < 0.001 and four asterisks (****) indicate a *p* value < 0.0001.

### Reporting summary

Further information on research design is available in the Nature Research Reporting Summary linked to this article.

### Supplementary information

Supplemental material

Reporting Summary

## Data Availability

The data discussed in this publication have been deposited in NCBI’s Gene Expression Omnibus and are accessible through GEO Series accession number GSE265869^65^.
