# Supplementary material for: Increased vascular smooth muscle cell senescence in aneurysmal Fibulin-4 mutant mice
Source: NPJ Aging. 2024 Jun 20;10(1):31. doi: 10.1038/s41514-024-00154-4 (PMC11189919; doi:10.1038/s41514-024-00154-4)
Supplement: Supplementary file 1 — Supplemental material [file 41514_2024_154_MOESM1_ESM.pdf]

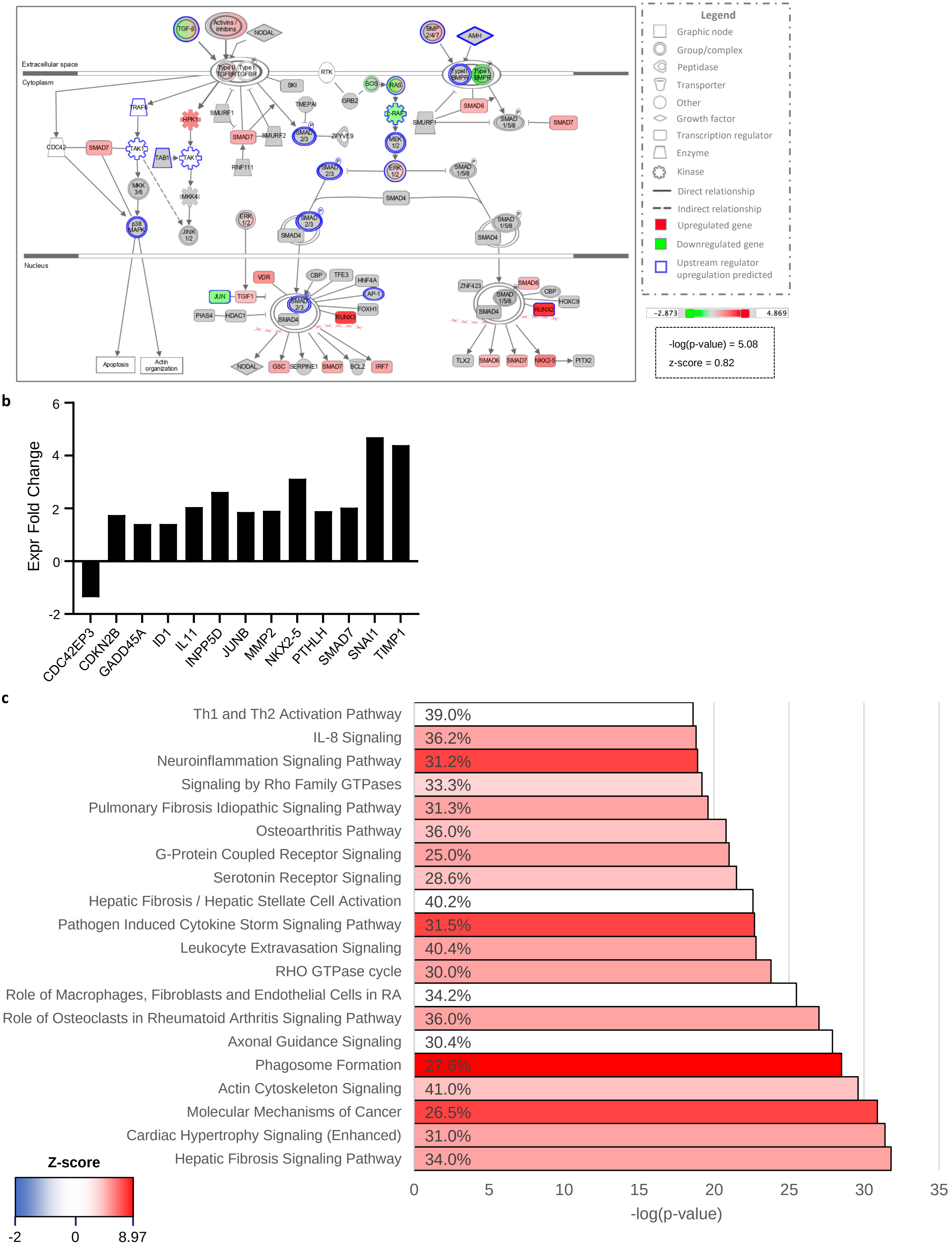

Supplementary Figure 1. IPA analysis of pathways of interest. **(a)** The TGF-β signaling pathway, adapted from IPA, portraying DEGs in the *Fibulin-4*$^{R/R}$ aortic arch compared to the *Fibulin-4*$^{+/+}$ aortic arch. Upregulated genes are indicated in red, downregulated genes are indicated in green and upstream regulators predicted to be upregulated have a blue outline. **(b)** Differential expression of genes downstream of the TGF-β signaling pathway in the Fibulin-4$^{R/R}$ aortic arch. **(c)** The top 20 significantly changed canonical pathways in the *Fibulin-4*$^{R/R}$ aortic arch.

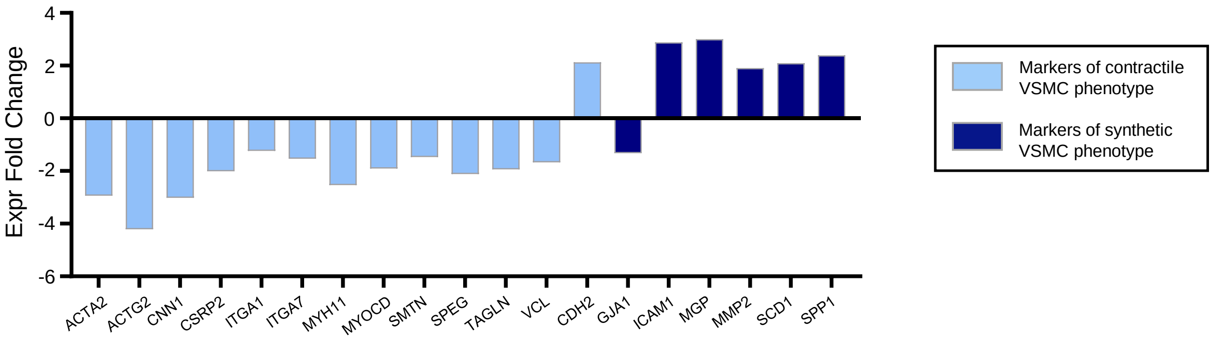

Supplementary Figure 2. Differential expression of genes that are markers for the contractile VSMC phenotype or the synthetic VSMC phenotype in the Fibulin-4$^{R/R}$ aortic arch.

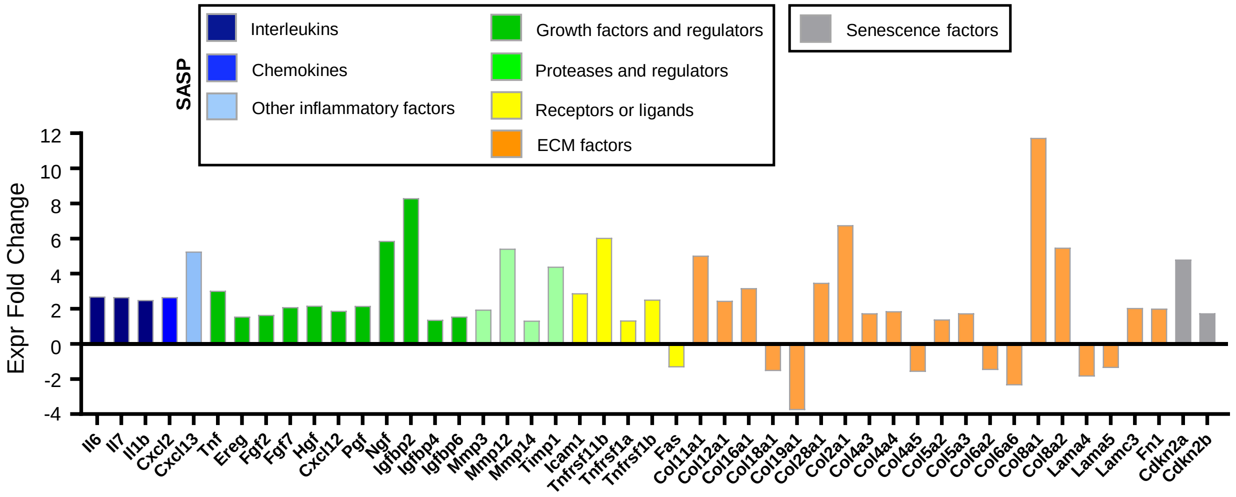

Supplementary Figure 3. Differential expression of SASP factors and senescence markers in the Fibulin-4$^{R/R}$ aortic arch.

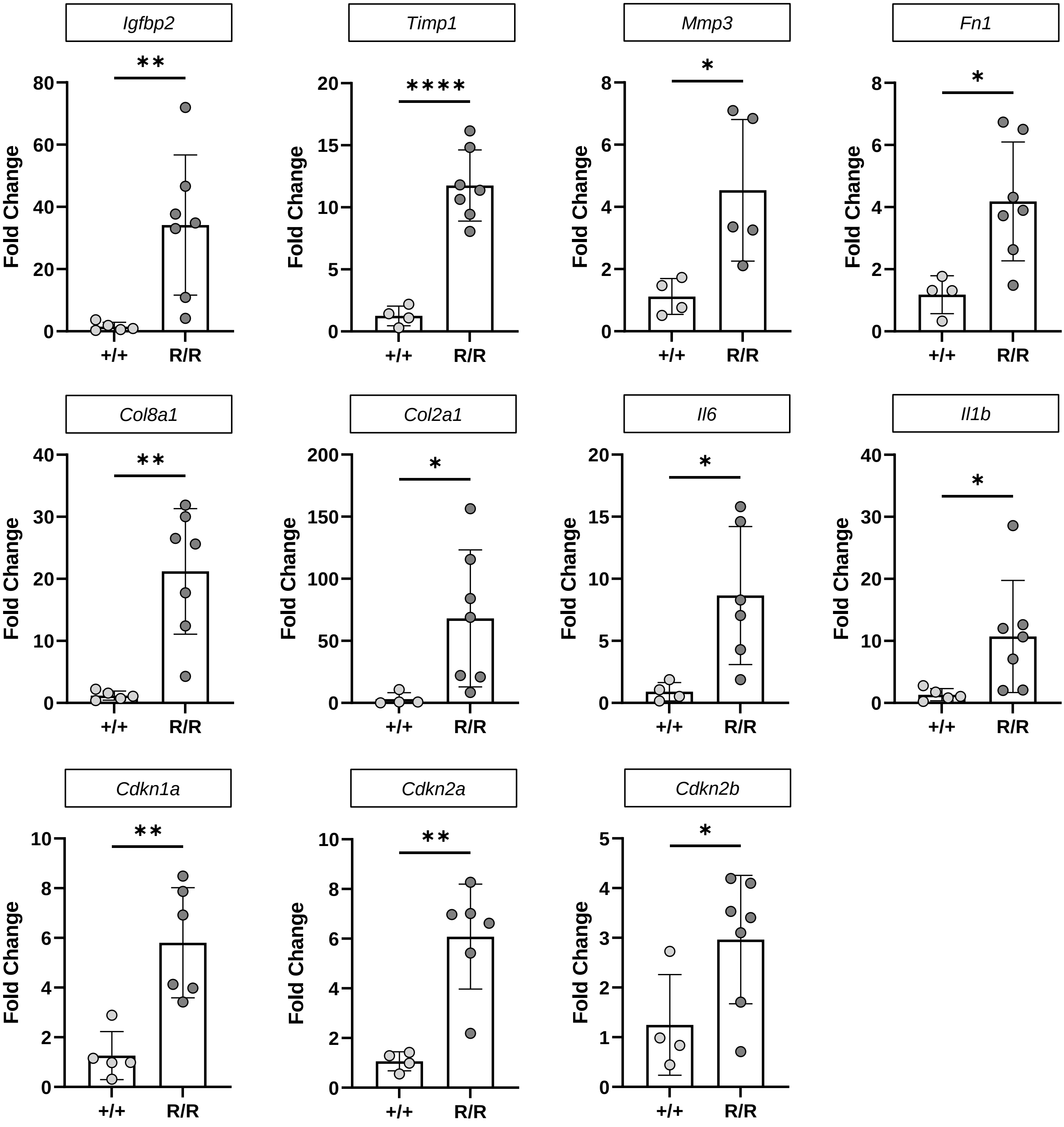

Supplementary Figure 4. qPCR of SASP factors in Fibulin-4$^{R/R}$ and Fibulin-4$^{+/+}$ aortic arch. qPCR data showing increased expression of SASP factors *Igfbp2, Timp1, Mmp3, Fn1, Col8a1, Col2a1, Il6, Il1b, Cdkn1a, Cdkn2a and Cdkn2b* in the *Fibulin-4$^{R/R}$* aortic arch compared to *Fibulin-4$^{+/+}$* controls (mean±SD are plotted, *p<0.05, **p<0.01, ****p<0.0001, unpaired t-test)

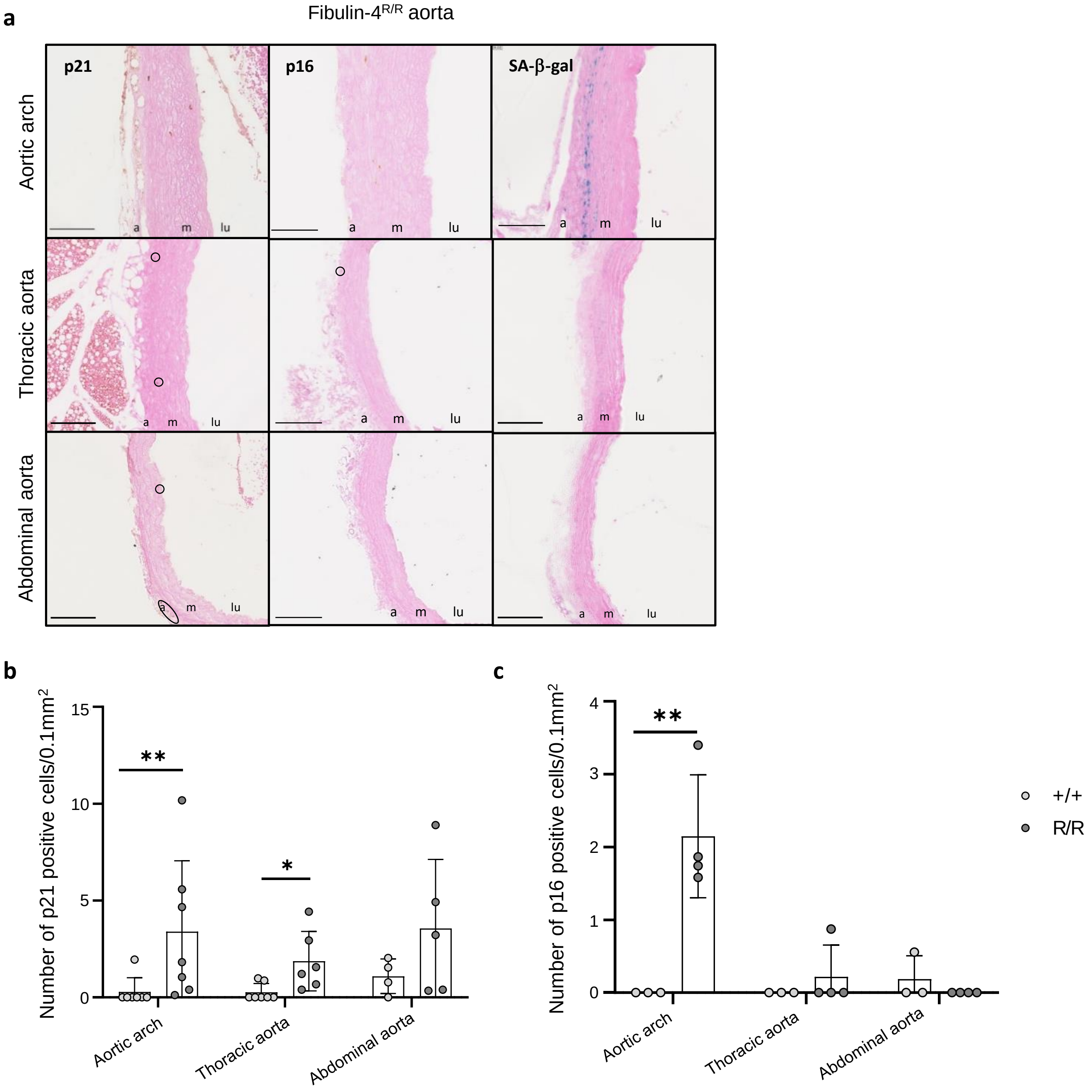

Supplementary Figure 5. Staining for senescence markers in Fibulin-4 mutant mouse aorta. **(a)** Representative images of immunohistochemical staining for p21, p16 and SA-β-gal in the different sections of the *Fibulin-4$^{R/R}$* aorta (aortic arch, thoracic aorta and abdominal aorta). **(b)** Quantification of immunohistochemical staining for p21 in *Fibulin-4$^{R/R}$* and *Fibulin-4$^{+/+}$* mouse aortas (n = 4-7 per group, *p<0.05, p**<0.01, unpaired t-test was performed for each location separately). **(c)** Quantification of immunohistochemical staining for p16 in *Fibulin-4$^{R/R}$* and *Fibulin-4$^{+/+}$* mouse aortas (n = 3-4 per group, p**<0.01, unpaired t-test was performed for each location separately).

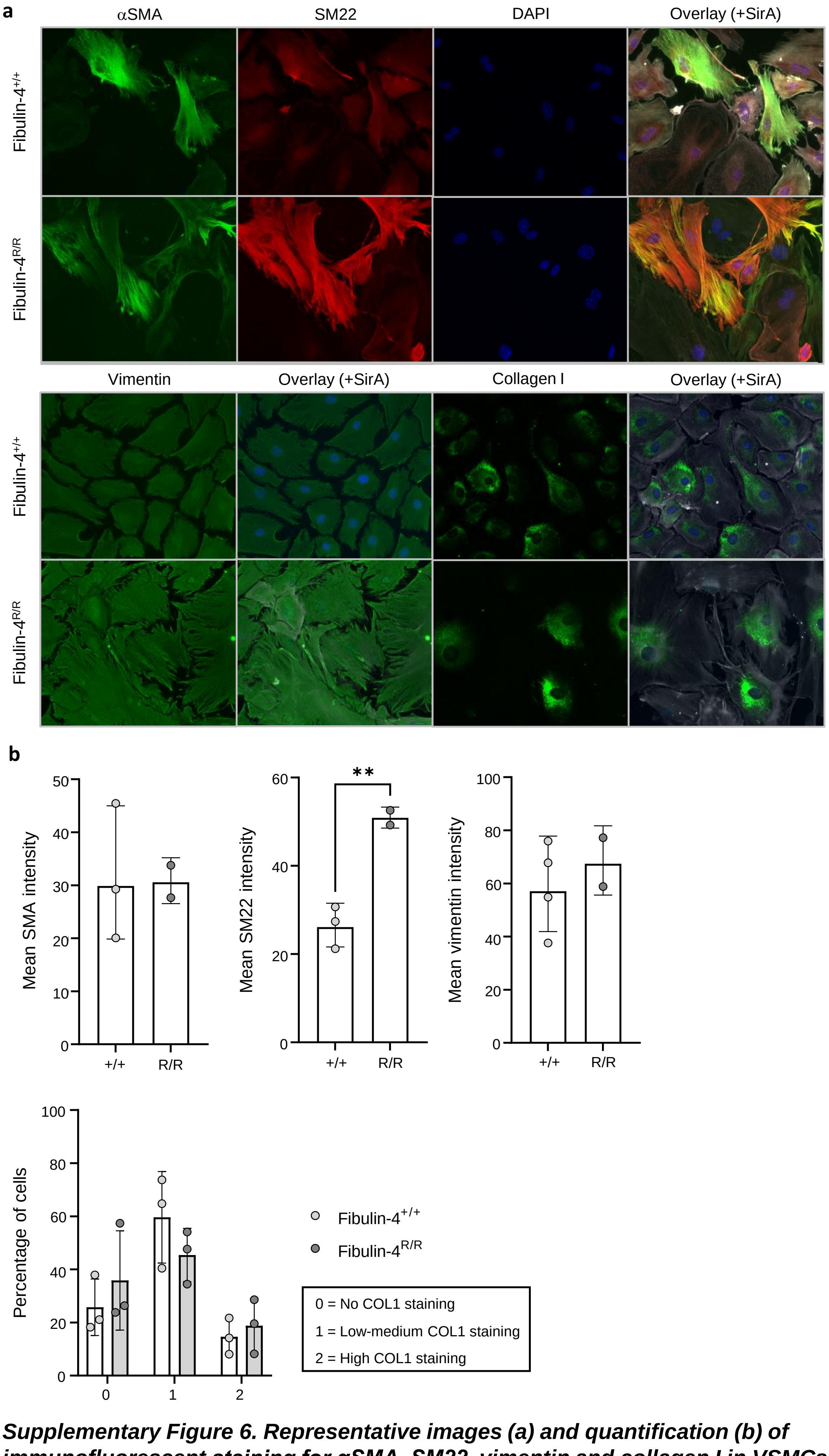

Supplementary Figure 6. Representative images (a) and quantification (b) of immunofluorescent staining for αSMA, SM22, vimentin and collagen I in VSMCs. The mean intensity (±SD) is plotted per cell line (n = 2-4 per group, **p<0.01, unpaired t-test).

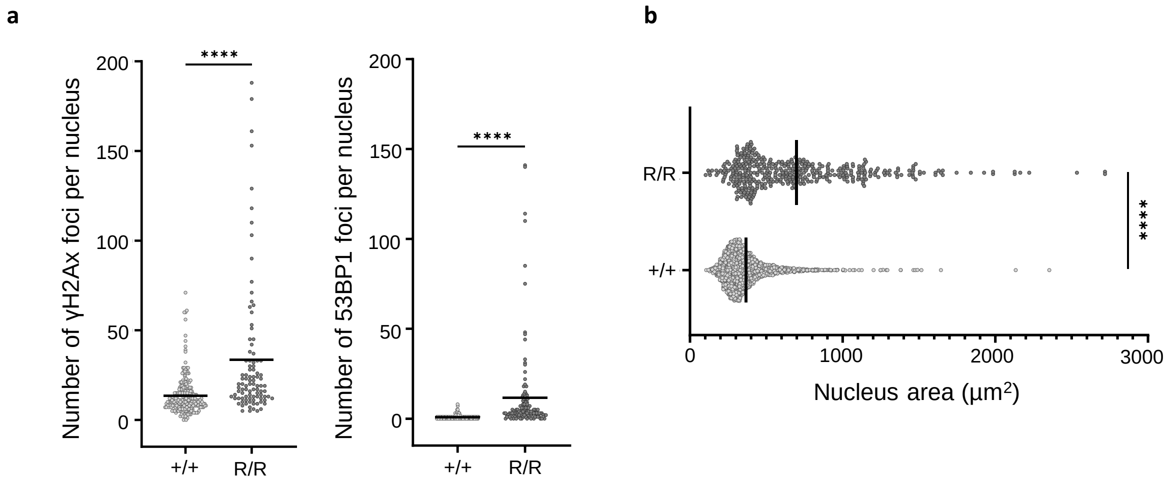

Supplementary Figure 7. Quantification of γH2Ax and 53BP1 foci in Fibulin-4 VSMCs in vitro **(a)** γH2Ax and 53BP1 foci quantification in *Fibulin-4$^{R/R}$* VSMCs and *Fibulin-4$^{+/+}$* controls. Each data point represents an individual nucleus, mean is plotted (n = 2-3 per group, p****<0.0001, Mann-Whitney test). **(b)** Quantification of the nucleus size (total nucleus area (µm^2)) of *Fibulin-4$^{R/R}$* VSMCs and *Fibulin-4$^{+/+}$* VSMCs. Each data point represents an individual nucleus, mean is plotted (n = 2-4 per group, p****<0.0001, Mann-Whitney test).

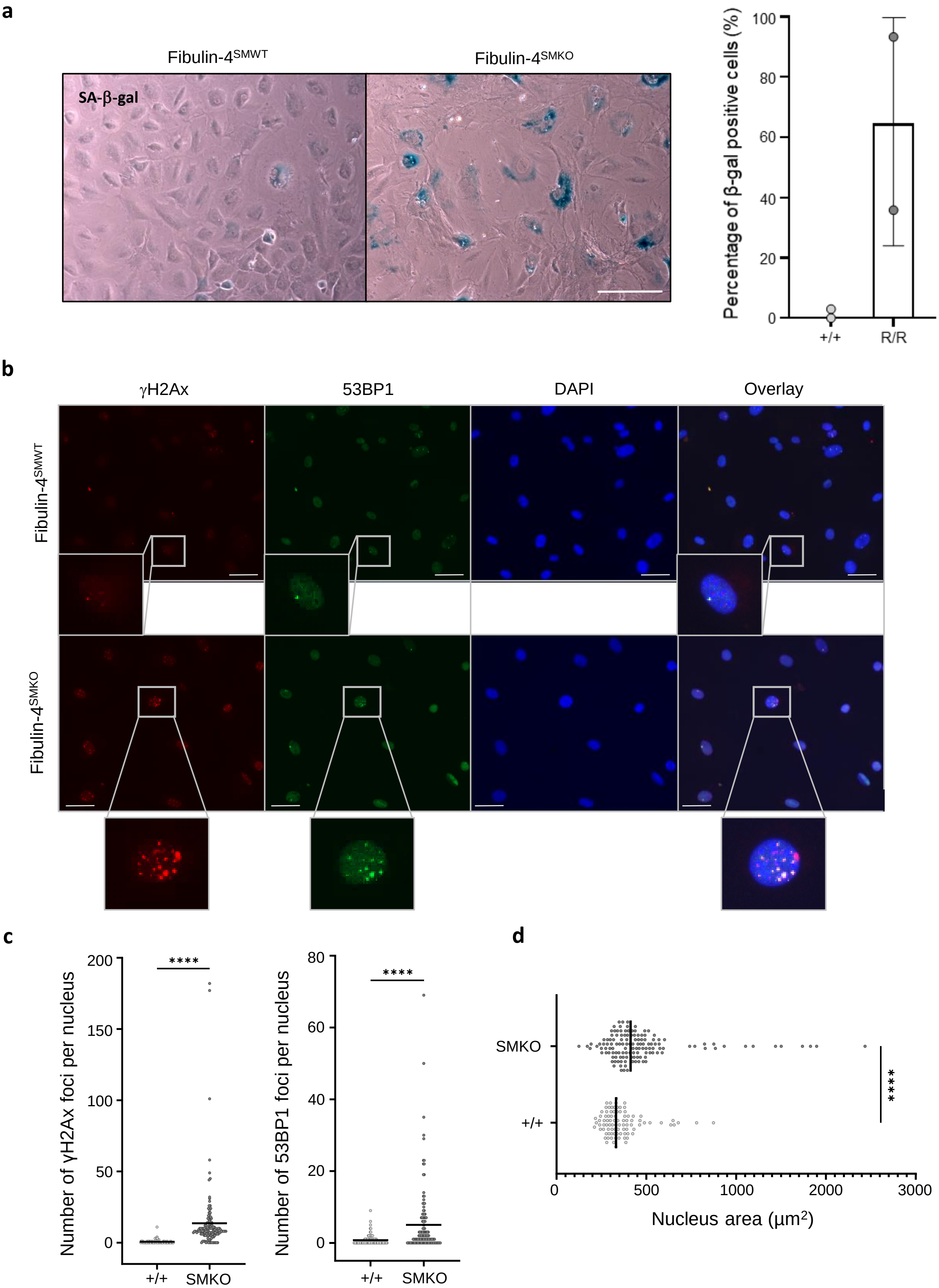

Supplementary Figure 8. Senescence markers SA-β-gal and DNA damage foci in Fibulin-4 SMKO VSMCs in vitro. **(a)** Representative images (left) and quantification (right) of SA-β-gal staining on *Fibulin-4^{SMKO}* VSMCs and *Fibulin-4$^{+/+}$* VSMCs. The mean (±SD) percentage of SA-β-gal positive cells are plotted (n = 2 per group, p = 0.333, Mann-Whitney test). *Bar = 500 µm.* **(b)** Representative images of immunofluorescent staining for DNA damage markers γH2Ax and 53BP1 on *Fibulin-4^{SMKO}* VSMCs and *Fibulin-4$^{+/+}$* VSMCs. *Bar = 50 µm.* **(c)** Quantification of the number of γH2Ax and 53BP1 foci per nucleus. Each data point represents an individual nucleus, mean is plotted (n = 1-3 per group, ****p<0.0001, Mann-Whitney test). **(d)** Quantification of the nucleus size (total nucleus area (µm^2)) of *Fibulin-4^{SMKO}* VSMCs and *Fibulin-4$^{+/+}$* VSMCs. Each data point represents an individual nucleus, mean is plotted (n = 1-3 per group, p****<0.0001, Mann-Whitney test).

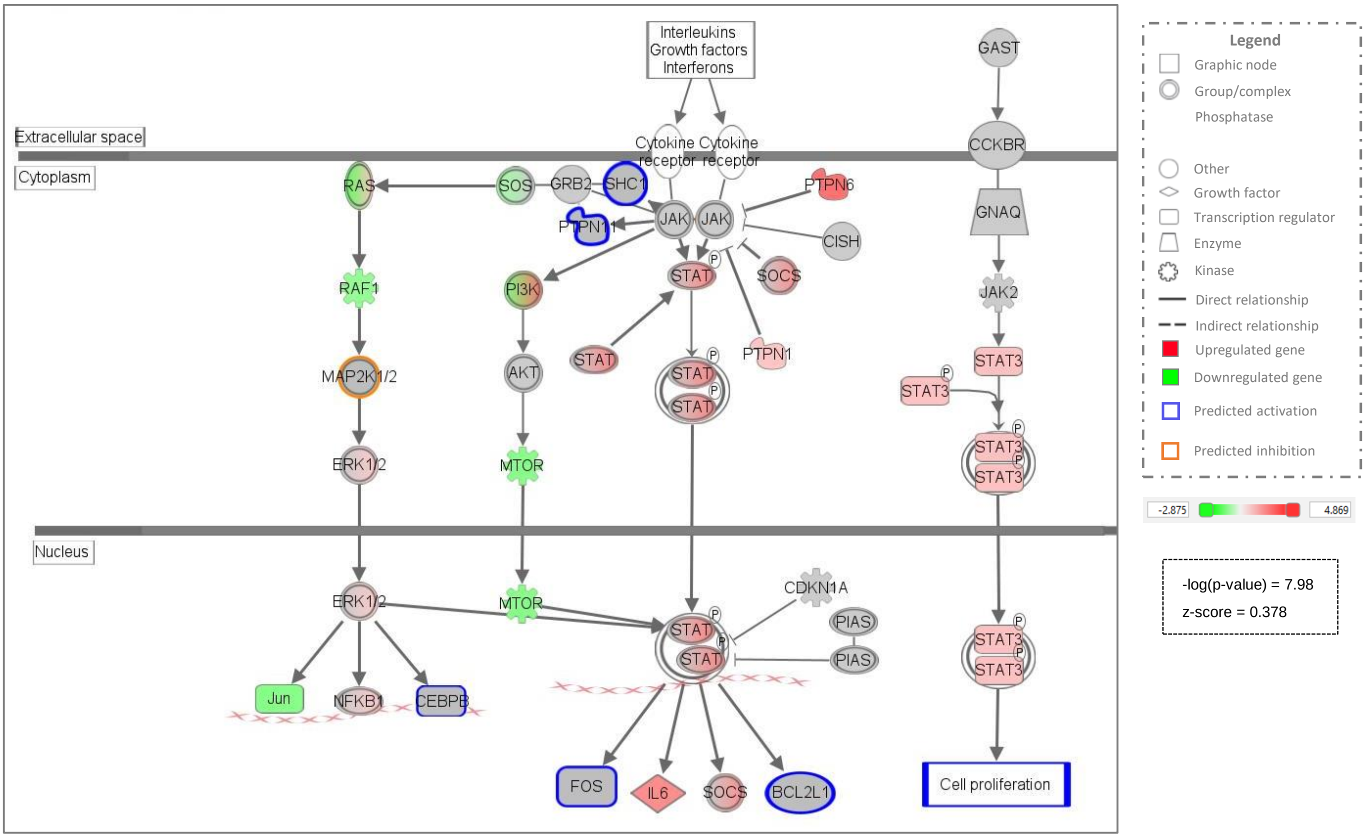

Supplementary Figure 9. IPA analysis of JAK/STAT pathway. The JAK/STAT signaling pathway, adapted from IPA, portraying DEGs in the *Fibulin-4*$^{R/R}$ aortic arch compared to the *Fibulin-4*$^{+/+}$ aortic arch. Upregulated genes are indicated in red, downregulated genes are indicated in green. Upstream regulators predicted to be upregulated have a blue outline and upstream regulators predicted to be inhibited have a orange outline.

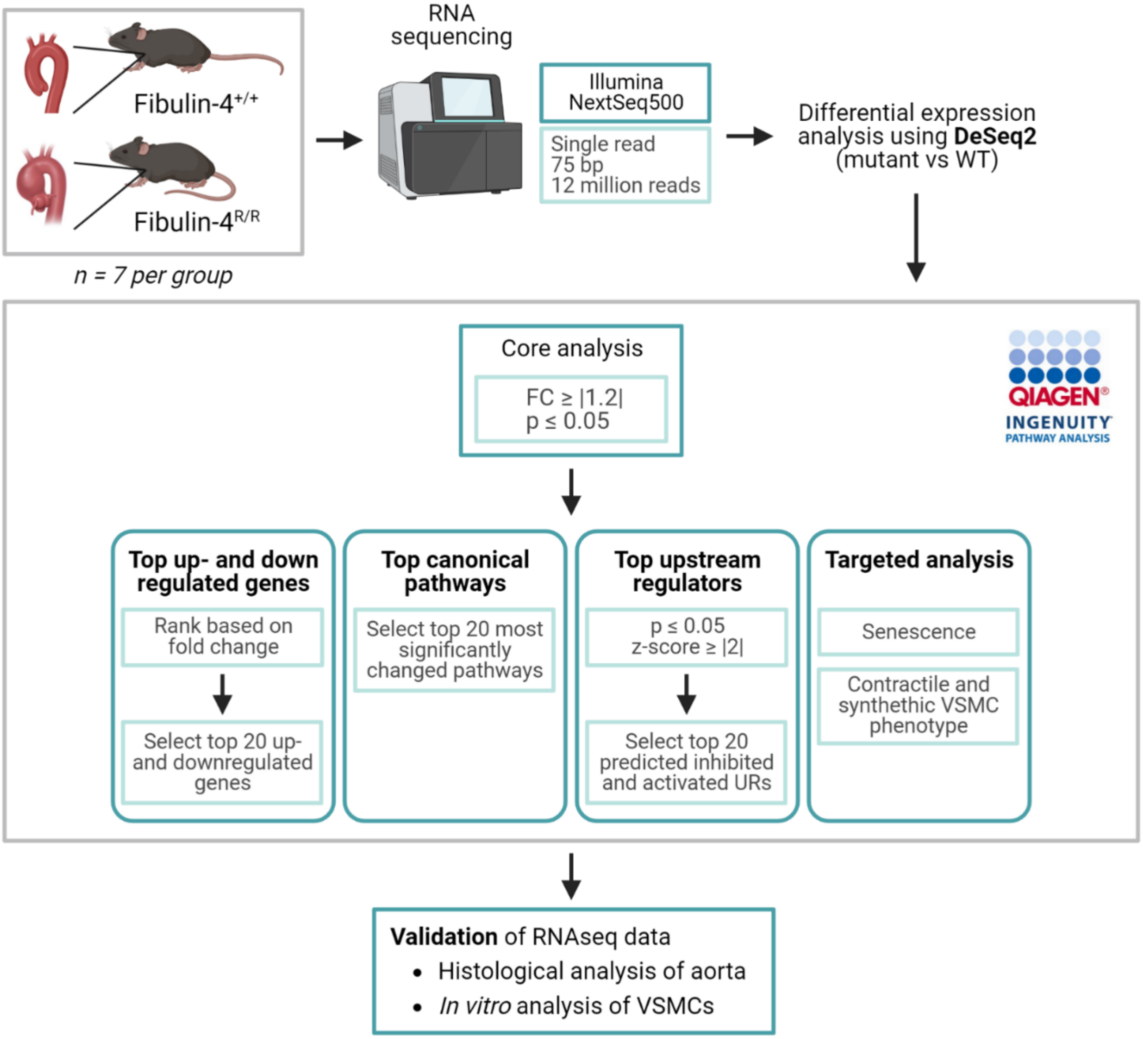

Supplementary Figure 10. Workflow of RNA sequencing experiment and subsequent data analysis.

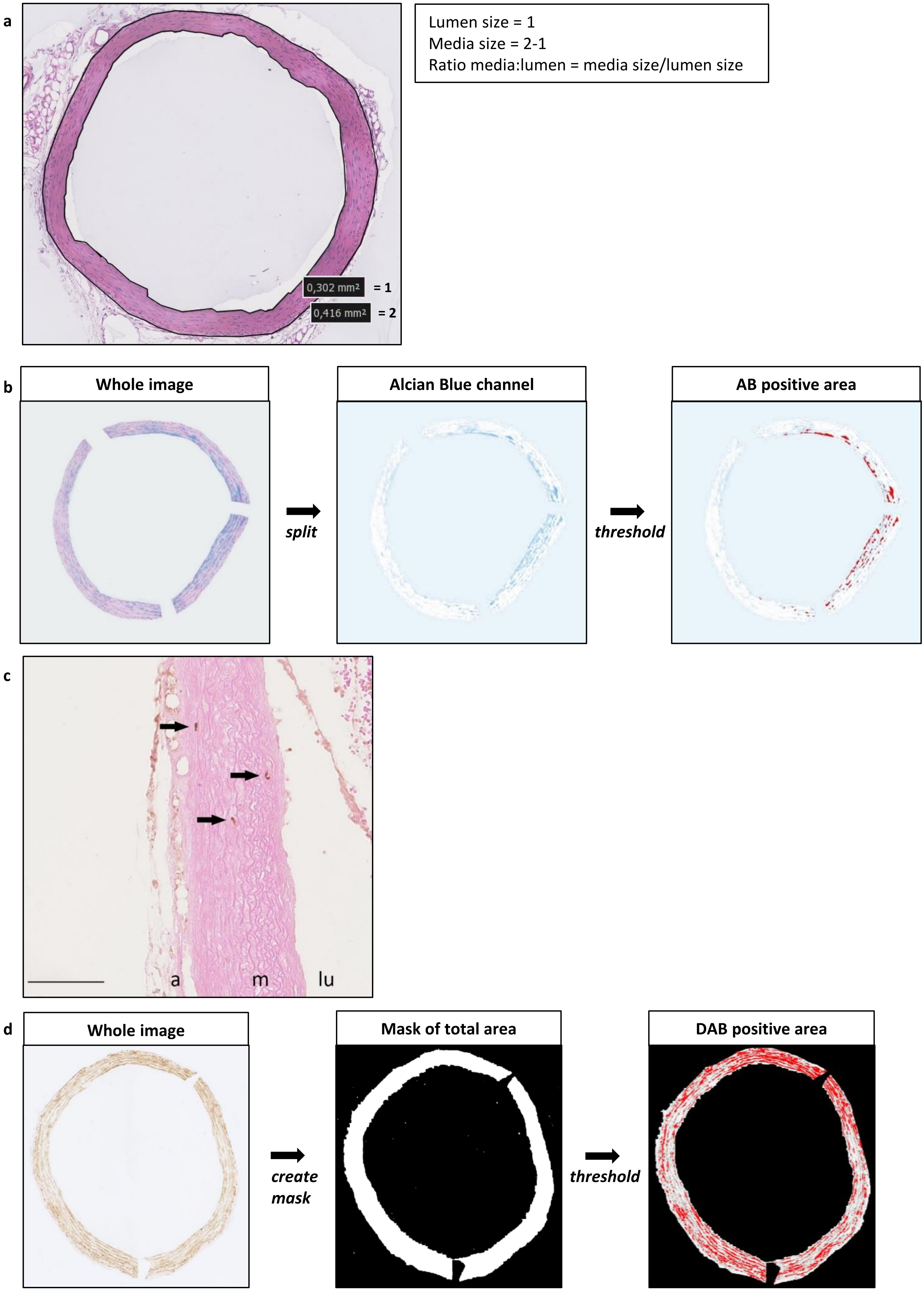
a
0,302 mm²
= 1
0,416 mm²
= 2
Lumen size = 1
Media size = 2-1
Ratio media:lumen = media size/lumen size
b
Whole image
split
Alcian Blue channel
threshold
AB positive area
c
a
m
lu
d
Whole image
create mask
Mask of total area
threshold
DAB positive area

Supplementary Figure 11. Quantification of (immuno)histochemical staining of mouse aorta. **(a)** Media and lumen size were determined on HE stained sections. Quantification was performed by manual selection of the media and lumen using the freehand region tool in NDP view.2 (Hamamatsu Photonics K.K., U12388-01), which calculates the surface area in mm^2. Formulas for the calculation of the lumen size, media size and the media:lumen ratio are shown. **(b)** Representative image of AB staining on the thoracic aorta of a Fibulin-$4^{R/R}$ mouse at 10x magnification. Parts of the aorta that were folded or out of focus were manually removed in ImageJ to ensure proper analysis. The whole image was split to obtain the ‘Alcian Blue’ channel. Subsequently, the threshold was set to ensure measurement of areas truly positive for AB staining. The percentage of AB positive area was calculated by dividing the positive area by the total area. **(c)** Representative image of p21 staining on the aortic arch of a Fibulin-$4^{R/R}$ mouse at 20x magnification. The cells that are counted positive for p21 are indicated with an arrow. The number of positive cells was corrected for media size. Media size was determined using the freehand region tool in NDP view.2 (Hamamatsu Photonics K.K., U12388-01), which calculates the surface area in mm^2. a = tunica adventitia, m = tunica media, lu = lumen **(d)** Representative images of αSMA staining on the thoracic aorta of a Fibulin-$4^{R/R}$ mouse at 10x magnification. Parts of the aorta that were folded or out of focus were manually removed in ImageJ to ensure proper analysis. The whole image was split to obtain the DAB channel. Subsequently, the threshold was set to ensure measurement of areas truly positive for αSMA staining. The αSMA density was calculated by dividing the positive area by the total area.

Supplementary Table 1. IPA core analysis settings and resulting number of analysis-ready genes per group.

**Experimental groups (comparisons)**		**Material**	**Fold Change cut-off**	**P-value cut-off**	**Number of DEGs after cut-off**
**Fibulin-4$^{R/R}$ mice**	Fibulin-4$^{+/+}$ mice	Aortic arch	$-1.2 \leq FC \geq 1.2$	$p \leq 0.05$	3036
**Fibulin-4$^{R/R}$ mice**	Fibulin-4$^{+/+}$ mice	Abdominal aorta	$-1.2 \leq FC \geq 1.2$	$p \leq 0.05$	851

Supplementary Table 2. Pathways involved in mechanosensing from IPA analysis of RNAseq data from Fibulin-4$^{R/R}$ mouse aortic arch.

Mechanosensing pathway	-log(p-value)	Ratio	Z-score	Significantly changed genes
**Integrin signaling**	18.5	0.358	0.372	ACTA2,ACTB,ACTC1,ACTG2,ACTN1,ACTN4,ARPC1B,BCAR3,CAV1,FYN,GSN,ILK,ITGA1,ITGA11,ITGA4,ITGA5,ITGA7,ITGA8,ITGA9,ITGAL,ITGAM,ITGAX,ITGB2,ITGB3,ITGB4,ITGB5,ITGB7,KRAS,MAPK3,MYL12A,MYL7,MYL9,MYLK,MYLK2,MYLK3,PAK2,PAK3,PFN1,PFN2,PIK3C2B,PIK3CA,PIK3CB,PIK3CD,PIK3CG,PIK3R5,PLCG1,PLCG2,PPP1CB,PPP1R12A,Ppp1r12b,PXN,RAC2,RAF1,RAP1B,RAP2A,RAP2B,RASD2,RHOBTB1,RHOD,RHOH,RHOT1,RND1,ROCK1,RRAS,SOS1,SRC,TLN1,TNK2,TSPAN2,TSPAN6,TSPAN7,TTN,VASP,VCL,WAS,ZYX
**ILK signaling**	17.6	0.358	1.524	ACTA2,ACTB,ACTC1,ACTG2,ACTN1,ACTN4,ATF2,ATF4,CCND1,CFL2,CREB5,FERMT2,FLNA,FLNC,FN1,GSK3A,HIF1A,ILK,IRS2,ITGB2,ITGB3,ITGB4,ITGB5,ITGB7,JUN,LIMS2,MAPK10,MAPK3,MTOR,MYC,MYH1,MYH11,MYH6,MYH7,MYH9,MYL3,MYL4,MYL6,MYL7,MYL9,MYO10,MYO18B,NFKB2,PDGFC,PGF,PIK3C2B,PIK3CA,PIK3CB,PIK3CD,PIK3CG,PIK3R5,PPP1R12A,PPP2CA,PXN,RAC2,RELB,RHOBTB1,RHOD,RHOH,RHOT1,RND1,RPS6KA5,RSU1,SNAI1,SNAI2,TESK1,TGFB1I1,TNF,TNFRSF1A,VCL,VEGFC,VIM
**Calcium signaling**	13	0.309	0.762	ACTA2,ACTC1,AKAP5,ASPH,ATF2,ATF4,ATP2B1,ATP2B4,CABIN1,CACNA1C,CACNA1E,CACNA1I,CACNA1S,CACNA2D1,CACNA2D2,CACNA2D3,CACNB2,CACNG4,CAMK1D,CAMK2A,CAMK2G,CAMK4,CASQ1,CASQ2,CREB5,GRIA1,GRIA3,HDAC10,HDAC5,HDAC9,ITPR1,ITPR3,MAPK3,MEF2D,MYH1,MYH11,MYH6,MYH7,MYH9,MYL3,MYL4,MYL6,MYL7,MYL9,MYO10,MYO18B,NFATC1,NFATC2,PNCK,PPP3CA,PPP3CC,PRKACB,PRKAR1B,PRKAR2A,RAP1B,RAP2A,RAP2B,RYR3,SLC8A2,TNNC1,TNNI3,TNNT2,TP63,Tpm1,Tpm2,TPM3,Tpm4,TRDN
**RhoA mediated signaling**	8.53	0.323	1.121	ACTA2,ACTB,ACTC1,ACTG2,ARHGAP4,ARHGAP9,ARPC1B,CDC42EP1,CDC42EP3,CFL2,CIT,EPHA1,EZR,LIMK1,LIMK2,LPAR2,LPAR5,MYL12A,MYL3,MYL4,MYL6,MYL7,MYL9,MYLK,MYLK2,MYLK3,NGEF,PFN1,PFN2,PIP4K2A,PIP4K2B,PIP5K1B,PIP5K1C,PPP1CB,PPP1R12A,Ppp1r12b,PTK2B,ROCK1,ROCK2,TTN
**Regulation of actin-based motility by Rho**	14.9	0.417	1.768	ACTA2,ACTB,ACTC1,ACTG2,ARPC1B,GSN,ITGA1,ITGA11,ITGA4,ITGA5,ITGA7,ITGA8,ITGA9,ITGAL,ITGAM,ITGAX,ITGB2,ITGB3,ITGB4,ITGB5,ITGB7,LIMK1,MYL12A,MYL3,MYL4,MYL6,MYL7,MYL9,MYLK,PAK2,PAK3,PFN1,PFN2,PIP4K2A,PIP4K2B,PIP5K1B,PIP5K1C,PPP1CB,PPP1R12A,Ppp1r12b,RAC2,RHOBTB1,RHOD,RHOH,RHOT1,RND1,ROCK1,WAS
**Actin cytoskeleton signaling**	29.6	0.41	2.321	ACTA2,ACTB,ACTC1,ACTG2,ACTN1,ACTN4,APC2,ARHGAP24,ARHGEF4,ARPC1B,CD14,CFL2,CSK,EZR,F2R,FGD3,FGF12,FGF13,FGF16,FGF18,FGF2,FGF23,FGF7,FLNA,FN1,GSN,IQGAP2,ITGA1,ITGA11,ITGA4,ITGA5,ITGA7,ITGA8,ITGA9,ITGAL,ITGAM,ITGAX,ITGB2,ITGB3,ITGB4,ITGB5,ITGB7,KRAS,LBP,LIMK1,LIMK2,MAPK3,MYH1,MYH11,MYH6,MYH7,MYH9,MYL12A,MYL3,MYL4,MYL6,MYL7,MYL9,MYLK,MYLK2,MYLK3,MYO10,MYO18B,NCKAP1L,PAK2,PAK3,PDGFC,PFN1,PFN2,PIK3C2B,PIK3CA,PIK3CB,PIK3CD,PIK3CG,PIK3R5,PIP5K1B,PIP5K1C,PPP1CB,PPP1R12A,Ppp1r12b,PXN,RAC2,RAF1,RAP1B,RAP2A,RAP2B,RASD2,ROCK1,ROCK2,RRAS,SOS1,SSH2,TIAM1,TLN1,TTN,VAV1,VAV2,VCL,WAS,WASF2
**FAK signaling**	4.57	0.163	6.749	ACKR4,ADORA2B,ADORA3,ADRA1D,ARPC1B,BCAR3,C3AR1,C5AR1,C5AR2,CALCRL,CCND1,CCR1,CCR2,CCR5,CCR7,CCR9,CD3D,CD3E,CD3G,CELSR1,CELSR2,CHRM2,CMKLR1,CNR2,COL18A1,COL2A1,COL5A3,CRHR2,CSF2RB,CSK,CX3CR1,CXCR2,CXCR6,CYSLTR1,ECM2,EDNRB,ELF4,ELK3,ETS1,ETV4,F2R,FCER1G,FLT4,FPR1,FYN,FZD2,FZD6,FZD7,FZD8,GALR2,GCGR,GPR132,GPR137B,GPR141,GPR153,GPR161,GPR174,GPR176,GPR18,GPR182,GPR183,GPR21,GPR34,GPR35,GPR37L1,GPR39,GPR65,GPR68,GPR85,GPR88,GPR89A/GPR89B,GRM1,GSK3A,HTR2B,IFNAR1,IFNLR1,IL10RA,IL10RB,IL13RA1,IL17RA,IL17RE,IL18R1,IL18RAP,IL1R1,IL1R2,IL1RL1,IL21R,IL22RA2,IL27RA,IL2RA,IL2RG,IL3RA,IL4R,IL7R,IL9R,ITGA1,ITGA11,ITGA4,ITGA5,ITGA7,ITGA8,ITGA9,ITGAL,ITGAM,ITGAX,ITGB2,ITGB3,ITGB4,ITGB5,ITGB7,JUN,KRAS,LCK,LGR4,LPAR2,LPAR5,LTB4R,MAPK10,MAPK11,MAPK3,MMP14,MMP2,MTOR,MYC,NFKB2,NPY1R,P2RY10,P2RY13,P2RY6,PAK2,PAK3,PIK3C2B,PIK3CA,PIK3CB,PIK3CD,PIK3CG,PIK3R5,PLCG1,PLCG2,PTGER4,PTGFR,PTGIR,PTPN12,PXN,RAF1,RAP1B,RAP2A,RAP2B,RASA1,RASD2,RRAS,S1PR1,S1PR3,SH2D2A,SOCS2,SOCS3,SOCS5,SOS1,SPARCL1,SRC,TBXA2R,TCF4,Tcf7,TGFB1,TGFB3,TGFBR2,USF1,VIPR2,WAS,XCR1

Supplementary Table 3. Genes expressed in VSMCs with a contractile or synthetic phenotype (from Rensen, SMM et al. (21)).

**Contractile phenotype**	
Gene	Protein
MYH11	SM-MHC
SMTN	Smoothelin
CNN1	SM-calponin
VCL	Meta-vinculin
ACTG2	Gamma-SMA
SPEG	APEG-1
CSRP2	CRP-2
ITGA1	Alpha-1 integrin
ITGB1	Beta-1 integrin
ITGA7	Alpha-7 integrin
MYOCD	Myocardin
CDH2	N-cadherin
CDH13	T-cadherin
ACTA2	Alpha-SMA
DES	Desmin
TAGLN	SM22-alpha
AEBP1	ACLP

**Synthetic phenotype**	
Gene	Protein
RBP1	CRBP-1
MYH10	Smemb
PDGFA	PDGF-A
SPP1	Osteopontin
ICAM1	ICAM-1
MGP	MGP
COL1A1	Collagen I
MMP2	Collagenase IV
CX43/GJA1	Connexin43
SDC1	Syndecan-1
SDC4	Syndecan-4
MSN	Moesin

Supplementary Table 4. Senescence-associated secretory phenotype (SASP) factors (from Coppé, JP et al.(22))

**Interleukins (IL)**	**Gene name**
IL-6	IL6
IL-7	IL7
IL-1a	IL1A
IL-1b	IL1B
IL-13	IL13
IL-15	IL15
**Chemokines (CXCL, CCL)**	
IL-8	CXCL8
GRO-a	CXCL1
GRO-b	CXCL2
GRO-g	CXCL3
MCP-2	CCL8
MCP-4	CCL13
MIP-1a	CCL3
MIP-3a	CCL20
HCC-4	CCL16
Eotaxin	CCL11
Eotaxin-3	CCL26
TECK	CCL25
ENA-78	CXCL5
I-309	CCL1
I-TAC	CXCL11
**Other inflammatory factors**	
GM-CSE	CSF2
G-CSE	CSF3
IFN-γ	IFNG
BLC	CXCL13
MIF	MIF
**Growth factors and regulators**	
Amphiregulin	AREG
Epiregulin	EREG
Heregulin	NRG1
EGF	EGF
bFGF	FGF2
HGF	HGF
KGF	FGF7
VEGF	VEGFA
Angiogenin	ANG
SCF	KITLG
SDF-1	CXCL12
PIGF	PGF
NGF	NGF
IGFBP-2	IGFBP-2
IGFBP-3	IGFBP-3
IGFBP-4	IGFBP-4
IGFBP-6	IGFBP-6
IGFBP-7	IGFBP-7

**Proteases and regulators**	**Gene name**
MMP-1	MMP1
MMP-3	MMP3
MMP-10	MMP10
MMP-12	MMP12
MMP-13	MMP13
MMP-14	MMP14
TIMP-1	TIMP1
TIMP-2	TIMP2
PAI-1	SERPINE1
PAI-2	SERPINB2
tPA	PLAT
mPA	PLAU
cathepsin B	CTSB
**Soluble or shed receptors or ligands**	
ICAM-1	ICAM1
ICAM-3	ICAM3
OPG	TNFRS11B
sTNFRI	TNFRS1A
TRAIL-R3	TNFRSF10C
Fas	FAS
sTNFRII	TNFRS1B
Fas ligand	FASLG
mPAR	
SGP130	IL6ST
EGF-R	EGFR
**Nonprotein soluble factors**	
PGE2	-
Nitric oxide	-
Reactive oxygen species	-
**Insoluble factors (ECM)**	
Fibronectin	FN1
Collagens	(Group)
Laminin	(Group)

Supplementary Table 5. Calculation of percentage of differentially expressed SASP factors.

Experimental groups (comparisons)		Material	Number of differentially expressed SASP factors	Percentage of total SASP
**Fibulin-4$^{R/R}$ mice**	Fibulin-4$^{+/+}$ mice	Aortic arch	28	(28/71)*100% = **39.43%**

Total amount of SASP factors reported by the literature source used is 70. We added TNF to this list resulting in a total of 71 SASP factors. Since there is no distinction between types of collagens or laminins, all differentially expressed genes encoding for different types of collagen chains or laminin subunits are each collectively counted as ‘1’ changed SASP factor. For example, in the Fibulin-4$^{R/R}$ aortic arch there are 16 differentially expressed collagen chains and 3 differentially expressed laminin subunits, so collectively these collagens and laminins were counted as 2 changed SASP factors.

Supplementary Table 6. qPCR primers

**Gene**	**Forward primer**	**Reverse primer**	**Tm**
Igfbp2	GCGGGTACCTGTGAAAAGAG	CCTCAGAGTGGTCGTCATCA	60 ºC
Timp1	TCGGACCTGGTCATAAGGGC	GCTTTCCATGACTGGGGTGT	55 ºC
Mmp3	TTCAGTCCCTCTATGGAACTCCC	AGCTGCACATTGGTGATGTC	60 ºC
Fn1	ACGGACATCTGTGGTGTAGC	CGAGTCTGAACCAAAACCGC	55 ºC
Col8a1	AGAGTACCCACACCTACCCC	GCTAACGGTACTTCTCCTTTGC	60 ºC
Col2a1	GCTGGTGAAGAAGGCAAACGAG	CCATCTTGACCTGGGAATCCAC	60 ºC
Il6	GGAGTCACAGAAGGAGTGGC	AACGCACTAGGTTTGCCGAG	60 ºC
Il1b	CACAGCAGCACATCAACAAG	GTGCTCATGTCCTCATCCTG	60 ºC
Cdkn1a	CAGAGGCCCAGTACTTCCT	CAATCTGCGCTTGGAGTGAT	60 ºC
Cdkn2a	ATCTGGAGCAGCATGGAGTC	CGAATCTGCACCGTAGTTGA	60 ºC
Cdkn2b	GGCAAGTGGAGACGGTG	CCATCATCATGACCTGGATTG	55 ºC
Hprt	TGTAATGATCAGTCAACGGG	ACAATCAAGACATTCTTTCCAG	55 ºC
Ppia	GTCTCCTTCGAGCTGTTTGC	ACCACCCTGGCACATGAATC	60 ºC

Supplementary Table 7. Protocol for enzymatic isolation of murine vascular smooth muscle cells

**Part**		**Explanation**
Materials		- 35 mm dishes<br>- 60 mm dishes<br>- 100 mm dishes<br>- 0,1% (w/v) Gelatin<br>- Sterile dissection material<br>- Collagenase type II (2 mg/ml in serum free DMEM: filter sterilized (0,2µm filter))<br>- PBS<br>- DMEM<br>- DMEM with 10% FCS and 1% Penicillin/streptomycin
Methods	*Coating dishes*	1. Dissolve 0,1% Gelatin (w/v) in MilliQ by autoclaving.<br>2. Add to a culture dish enough volume of 0,1% gelatin to cover the bottom.<br>3. Leave it for at least 15-30 min at room temperature.<br>4. Remove the gelatin from the dishes and use them for tissue culturing.
	Isolating aorta	1. Anesthetize the animal and clean chest and abdomen with 70% ethanol.<br>2. Open the midline of the abdomen and open the thorax to expose heart and lungs<br>3. Perfuse the heart via the left ventricle with PBS.<br>4. Remove the aorta from the animal<br>5. Dissect the fat and/or connective tissue with forceps under a stereoscopic microscope. When isolating also abdominal smooth muscle cells, separate abdominal part of the aorta from thoracic part.
	Enzymatic digestion	1. Split thoracic aorta in 2 pieces, arch (after left subclavian artery) and descending aorta.<br>2. Cut the aorta pieces in 1-2 mm pieces and transfer to a small culture tube.<br>3. Add enough DMEM| Collagenase type II to the tube to cover the aorta pieces.<br>4. Incubate aorta pieces at 37°C, 5% CO2 for 1h- 6h in the rotor, until aorta pieces look like they are dissolved. (exact time need to be determined)<br>5. Transfer cell suspension to a 15 ml falcon tube and dilute to cell suspension to a total volume of 3ml.<br>6. Concentrate cells by centrifugation at 1000G for 5 min. Remove the supernatant.<br>7. Resuspend the pellet in 0,75 ml DMEM with 10% FCS and 1% Pen/Strep and plate in 35 mm dish.<br>8. Let the cells grow. (don't refresh the medium until the 4th day of culture)
	Expanding VSMCs	1. When the 35 mm dish is 80-90% confluent with cells they are ready for expanding.<br>2. Remove the medium and wash the dish gently 2X with PBS.<br>3. Add Trypsin/EDTA for detaching the SMC.<br>4. When the cells are detached, resuspend the cells in medium and transfer them to a 60 mm gelatinized dish. (◊ passage 1)<br>5. If the 60 mm dish is confluent for 80-90%, split the cells into 10cm gelatinized dishes (◊ passage 2).<br>6. If the 10 cm dish is confluent for 80-90%, split the cells into 4 new 10cm gelatinized dishes (◊ passage 3).<br>7. When passage 3 dishes are confluent, there are enough cells to make vials for LN2 storage. Normally 1 vial per plate.

Supplementary Table 8. Primary and secondary antibodies used for immunofluorescent staining of VSMCs.

Primary antibody	Dilution	Manufacturer	Catalog number	Secondary antibody	Dilution	Manufacturer
**Mouse α-SMA**	1:750	Abcam	Ab7818	**α-mouse alexa 488**	1:1000	Molecular Probes
**Rabbit α-SM22**	1:400	Abcam	Ab14106	**α-rabbit alexa 594**	1:1000	Molecular Probes
**Rabbit α-vimentin**	1:750	Abcam	Ab2547	**α-rabbit alexa 488**	1:1000	Molecular Probes
**Rabbit α-collagen I**	1:100	Abcam	Ab21286	**α-rabbit alexa 488**	1:1000	Molecular Probes
**Rat α-p21**	1:100	Abcam	Ab107099	**α-rat alexa 594**	1:1000	Molecular Probes
**Rabbit α-53BP1**	1:1000	Novus Biologicals	NB100-904	**α-rabbit alexa 488**	1:1000	Molecular Probes
**Mouse α-γH2AX**	1:1000	Sigma Aldrich	05-636	**α-mouse alexa 594**	1:1000	Molecular Probes
